# The incidence and antimicrobial resistance of *Shigella*-attributable diarrhoea in young children in low-income and middle-income countries from the multicountry Enterics for Global Health (EFGH) *Shigella* Surveillance Study: a prospective, facility-based hybrid surveillance study

**DOI:** 10.1016/S2214-109X(25)00534-0

**Published:** 2026-03-11

**Authors:** Mohammad Tahir Yousafzai, Jennifer Cornick, Pablo Penataro Yori, M Jahangir Hossain, Adama Mamby Keita, Hannah E Atlas, Farhana Khanam, Richard Omore, Sean R Galagan, Naveed Ahmed, Faisal Ahmmed, Alex O Awuor, Henry Badji, Bakary Conteh, Maria Garcia Quesada, Paul F Garcia-Bardales, Bri'Anna Horne, Aneeta Hotwani, Eric R Houpt, Md Taufiqul Islam, Khuzwayo C Jere, Jane Juma, Jie Liu, Donnie Mategula, Billy Ogwel, Caleb Okonji, Uduma Uma Onwuchekwa, Maribel Paredes Olortegui, James A Platts-Mills, Sonia Qureshi, Md Nazmul Hasan Rajib, Elizabeth T Rogawski McQuade, Francesca Schiaffino, Ousman Secka, Samba O Sow, Desiree Witte, Karen L Kotloff, Nigel A Cunliffe, John D Clemens, Sharon M Tennant, Farah Naz Qamar, Margaret N Kosek, Patricia B Pavlinac, Milagritos D Tapia, John Benjamin Ochieng, EFGH Consortium, Isaiah Akello, Ethel Alumando, Manase Amolloh, Raphael Anyango, Md Taufiqur Rahman Bhuiyan, Bubacarr E. Ceesay, Umberto D'Alessandro, Ryan Dodd, Sarah E. Elwood, Irum Fatima, Erika Feutz, Md Ismail Hossen, Mahzabeen Ireen, Samba Juma Jallow, Sheikh Jarju, Mehrab Karim, Youssouf Keita, Zubair Latif, Clement Lefu, Anya M. Lewin, Rebecca Maguire, Katia Manzanares Villanueva, Christine J. McGrath, Arianna Rubin Means, Chloe Morozoff, Stephen Munga, Vitumbiko Yagontha Munthali, Maureen Ndalama, Latif Ndeketa, Caren Oreso, Tackeshy Pinedo Vasquez, Firdausi Qadri, Syed Qudrat-E-Khuda, Lucero Romaina-Cachique, Queen Saidi, Doh Sanogo, Olivia Lang Schultes, Wagner Valentino Shapiama Lopez, Kirkby D. Tickell, Moussa Traore, Loyda Fiorella Zegarra Paredes

**Affiliations:** a Department of Pediatrics and Child Health, Aga Khan University, Karachi, Pakistan; b School of Population Health, The University of New South Wales Sydney, Kensington, NSW, Australia; c Institute of Infection, Veterinary and Ecological Sciences, Faculty of Health and Life Sciences, University of Liverpool, Liverpool, UK; d Malawi Liverpool Wellcome Trust Research Programme, Blantyre, Malawi; e Division of Infectious Diseases and International Health, University of Virginia, Charlottesville, VA, USA; f Asociacion Benefica Prisma, Iquitos, Loreto, Peru; g Medical Research Council Unit The Gambia at the London School of Hygiene & Tropical Medicine, Banjul, The Gambia; h Center for Vaccine Development Mali, Bamako, Mali; i Department of Global Health, University of Washington, Seattle, WA, USA; j International Centre for Diarrhoeal Disease Research, Bangladesh, Dhaka, Bangladesh; k Center for Global Health Research, Kenya Medical Research Institute, Kisumu, Kenya; l Department of Epidemiology, Emory University, Atlanta, GA, USA; m Center for Vaccine Development and Global Health, University of Maryland School of Medicine, Baltimore, MD, USA; n Department of Medical Laboratory Sciences, School of Life Sciences and Health Professions, Kamuzu University of Health Sciences, Blantyre, Malawi; o School of Public Health, Qingdao University, Qingdao, Shandong, China; p Liverpool School of Tropical Medicine, Liverpool, UK; q Facultad de Medicina Veterinaria y Zootecnia, Universidad Peruana Cayetano Heredia, Lima, Peru; r International Vaccine Institute, Seoul, South Korea; s Department of Epidemiology, Fielding School of Public Health, University of California, Los Angeles, CA, USA; t Department of Medicine, University of Maryland School of Medicine, Baltimore, MD, USA; u Department of Epidemiology, University of Washington, Seattle, WA, USA

## Abstract

**Background:**

*Shigella* is a leading cause of dysentery and watery diarrhoea in low-income and middle-income countries (LMICs) with consequences beyond diarrhoea for children younger than 5 years, including environmental enteric dysfunction and linear growth impairment. We established the burden, serotypes, and antibiotic resistance patterns of *Shigella*-diarrhoea among young children in LMICs to inform vaccine trial planning and eventual vaccine introduction in high-burden countries.

**Methods:**

The Enterics for Global Health (EFGH) study was a prospective, facility-based hybrid surveillance study conducted from June 21, 2022, to Aug 25, 2024, across seven countries: Kenya, Malawi, Mali, The Gambia, Bangladesh, Pakistan, and Peru. Children aged 6–35 months presenting at selected health-care facilities with acute diarrhoea (three or more abnormally loose or watery stools in the last 24-h period lasting less than 14 days) were enrolled. We calculated care-seeking adjusted incidence estimates from contemporaneous population enumeration and by ascertaining health-care seeking patterns from a health-care utilisation survey conducted in children aged 6–35 months in the health-facility catchment area. We deemed *Shigella* to be attributable if detected by microbiological culture or by quantitative PCR (qPCR) using an *ipaH* quantification cycle threshold of less than or equal to 29·5 from rectal swabs. We determined antimicrobial resistance to commonly used antibiotics by disc diffusion. We calculated adjusted incidence for all participating country sites and by key subgroups of interest (age, diarrhoea severity, study month, *Shigella* species, and serotype).

**Findings:**

Of the 9476 enrolled children, 4316 (45·5%) were female and 5160 (54·5%) were male, 881 (9·3%) had *Shigella* detected by culture and 1870 (20·0%) by qPCR (among 9354 children with qPCR results available). *Shigella flexneri* dominated (497 [56·2%] of 881 by culture and 756 [39·4%] of 1870 by qPCR), with *S flexneri* 2a and *S flexneri* 6 being the most common serotypes by both methods. Across study sites, the adjusted incidences of *Shigella*-attributed diarrhoea by culture ranged from 2·7 per 100 child-years (95% CI 1·9–4·3) in Malawi to 11·7 per 100 child-years (8·3–24·2) in Peru and by qPCR ranged from 3·5 per 100 child-years (2·5–5·4) in Malawi to 26·9 per 100 child-years (19·0–40·9) in The Gambia. *Shigella* isolates exhibited resistance to WHO-recommended antibiotics for dysentery with variability across sites: ciprofloxacin (37·2% [range 14·0–74·0]), azithromycin (22·1% [1·2–34·2]), and ceftriaxone (16·2% [0·0–64·4]).

**Interpretation:**

*Shigella*-attributed diarrhoea is common among young children in LMICs, with its escalating antimicrobial resistance posing a serious threat to global public health. The leading quadrivalent vaccine candidates cover the majority of *Shigella* serotypes identified in this study. These data affirm both the need for *Shigella* vaccines and readiness of EFGH sites to conduct rigorous vaccine trials.

**Funding:**

The Gates Foundation.

## Résumé

Contexte : Shigella est une cause majeure de dysenterie et de diarrhée aqueuse dans les pays à revenu faible et intermédiaire (PRFI), avec des conséquences au-delà de la diarrhée chez les enfants de moins de 5 ans, y compris la dysfonction entérique environnementale et l’altération de la croissance linéaire. Nous avons étudié le fardeau, les sérotypes et les profils de résistance aux antibiotiques de la diarrhée à *Shigella* chez les jeunes enfants dans les PRFI afin d’informer la planification des essais de vaccins et l’introduction éventuelle du vaccin dans les pays à forte charge.

Méthodes : L’étude Enterics for Global Health (EFGH) était une étude de surveillance prospective, hybride et basée dans des établissements, menée du 21 juin 2022 au 25 août 2024 dans sept pays : Kenya, Malawi, Mali, Gambie, Bangladesh, Pakistan et Pérou. Des enfants âgés de 6 à 35 mois présentant une diarrhée aiguë (trois selles anormalement molles ou aqueuses ou plus au cours des dernières 24 heures, durant moins de 14 jours) dans des établissements de santé sélectionnés ont été inclus. Nous avons calculé l’incidence ajustée à la recherche de soins. estimations provenant du recensement de la population contemporain et en déterminant les comportements de recherche de soins de santé à partir d’une enquête d’utilisation des soins de santé menée auprès d’enfants âgés de 6 à 35 mois dans la zone de desserte des établissements de santé. Nous avons considéré que Shigella était imputable si détectée par culture microbiologique ou par PCR quantitative (qPCR) en utilisant un seuil de cycle de quantification ipaH inférieur ou égal à 29,5 à partir d’échantillons rectaux. Nous avons déterminé la résistance aux antimicrobiens pour les antibiotiques couramment utilisés par diffusion sur disque. Nous avons calculé l’incidence ajustée pour tous les sites de pays participants et par sous-groupes clés d’intérêt (âge, gravité de la diarrhée, mois de l’étude, espèces de *Shigella* et sérotype).

Résultats : Parmi les 9476 enfants inclus, 4316 (45,5 %) étaient des filles et 5160 (54,5 %) des garçons, 881 (9,3 %) avaient Shigella détectée par culture et 1870 (20,0 %) par qPCR (parmi 9354 enfants pour lesquels les résultats de la qPCR étaient disponibles). *Shigella flexneri* dominait (497 [56,2 %] sur 881 par culture et 756 [39,4 %] sur 1870 par qPCR), avec *S. flexneri 2a* et *S. flexneri 6* étant les sérotypes les plus courants selon les deux méthodes. Dans l’ensemble des sites d’étude, les incidences ajustées de diarrhée attribuable à *Shigella* selon la culture variaient de 2,7 pour 100 années-enfants (IC 95 % 1,9–4,3) au Malawi à 11,7 pour 100 années-enfants (8,3–24,2) au Pérou, et selon la qPCR, elles variaient de 3,5 pour 100 années-enfants (2,5–5,4) au Malawi à 26,9 pour 100 années-enfants (19,0–40,9) en Gambie. Les isolats de *Shigella* présentaient une résistance aux antibiotiques recommandés par l’OMS pour la dysenterie avec une variabilité selon les sites: ciprofloxacine (37,2 % [plage 14,0–74,0]), azithromycine (22,1 % [1,2–34,2]) et ceftriaxone (16,2 % [0,0–64,4]).

Interprétation : la diarrhée attribuable à *Shigella* est fréquente chez les jeunes enfants dans les PMA, et la résistance antimicrobienne croissante constitue une menace sérieuse pour la santé publique mondiale. Les principaux candidats vaccins quadrivalents couvrent la majorité des sérotypes de Shigella identifiés dans cette étude. Collectivement, ces données confirment à la fois la nécessité et la préparation des sites EFGH à forte charge à mener des essais cliniques rigoureux sur les vaccins contre la *Shigella*.

## Abstract (es)

**Antecedentes.***Shigella* es una de las principales causas de disentería y diarrea acuosa en países de ingresos bajos y medianos (PIBM), con consecuencias que van más allá de la diarrea en niños menores de 5 años, incluyendo disfunción entérica ambiental y retraso en el crecimiento lineal. Determinamos la carga, los serotipos y los patrones de resistencia a antibióticos de la diarrea por *Shigella* en niños pequeños en PIBM, con el fin de orientar la planificación de ensayos clínicos de vacunas y la eventual introducción de una vacuna en países con alta carga de enfermedad.

**Métodos.** El estudio Enterics for Global Health (EFGH) fue un estudio de vigilancia híbrido, prospectivo y basado en establecimientos de salud, realizado entre el 21 de junio de 2022 y el 25 de agosto de 2024 en siete países: Kenia, Malaui, Malí, Gambia, Bangladesh, Pakistán y Perú. Se enrolaron niños de 6 a 35 meses que acudieron a establecimientos de salud seleccionados presentando un cuadro de diarrea aguda (definida como tres o más deposiciones anormalmente blandas o acuosas en un período de 24 horas, con una duración menor de 14 días). Calculamos estimaciones de incidencia ajustadas por búsqueda de atención médica utilizando una enumeración poblacional contemporánea y determinando los patrones de búsqueda de atención mediante una encuesta de utilización de servicios de salud realizada en niños de 6 a 35 meses en el área de captación de cada establecimiento. Consideramos que la diarrea era atribuible a *Shigella* cuando se detectó mediante cultivo microbiológico o por PCR cuantitativa (qPCR) a partir de hisopos rectales, utilizando un umbral de ciclo de cuantificación para el gen ipaH menor o igual a 29,5. La resistencia antimicrobiana a antibióticos de uso común se determinó mediante difusión en disco. Calculamos incidencias ajustadas para todos los sitios participantes, así como por subgrupos clave de interés, incluyendo edad, gravedad de la diarrea, mes de estudio, especie de *Shigella* y serotipo.

**Resultados.** De los 9476 niños inscritos, 4316 (45,5%) eran mujeres y 5160 (54,5%) hombres. *Shigella* fue detectada en 881 (9,3%) niños mediante cultivo y en 1870 (20,0%) mediante qPCR (entre 9354 niños con resultados de qPCR disponibles). *Shigella flexneri* fue la especie predominante (497 [56,2%] de 881 por cultivo y 756 [39,4%] de 1870 por qPCR), siendo los serotipos más frecuentes *S. flexneri* 2a y *S. flexneri* 6 según ambos métodos. Entre los diferentes sitios del estudio, las incidencias ajustadas de diarrea atribuida a *Shigella* por cultivo oscilaron entre 2,7 por 100 años-niño (IC 95%: 1,9–4,3) en Malaui y 11,7 por 100 años-niño (IC 95%: 8,3–24,2) en Perú, mientras que las estimaciones por qPCR oscilaron entre 3,5 por 100 años-niño (IC 95%: 2,5–5,4) en Malaui y 26,9 por 100 años-niño (IC 95%: 19,0–40,9) en Gambia. Los aislamientos de *Shigella* mostraron resistencia a antibióticos recomendados por la OMS para el tratamiento de la disentería, con variabilidad entre sitios: ciprofloxacino (37,2% [rango 14,0–74,0]), azitromicina (22,1% [1,2–34,2]) y ceftriaxona (16,2% [0,0–64,4]).

**Interpretación.** La diarrea atribuida a *Shigella* es frecuente en niños pequeños en países de ingresos bajos y medianos, y el aumento de la resistencia antimicrobiana representa una amenaza significativa para la salud pública mundial. Los principales candidatos a vacunas cuadrivalentes cubren la mayoría de los serotipos identificados en este estudio. En conjunto, estos hallazgos confirman tanto la necesidad como la capacidad de los sitios con alta carga del estudio EFGH para llevar a cabo ensayos clínicos rigurosos de vacunas contra *Shigella*.

## Abstract (ur)

خلاصہ پس منظر۔ شیجیلا کم آمدنی اور درمیانی آمدنی والے ممالک (LMICs) میں ڈسینٹری اور پانی والی اسہال کا ایک اہم سبب ہے، جس کے اثرات صرف اسہال تک محدود نہیں ہیں بلکہ پانچ سال سے کم عمر بچوں میں ماحولیاتی آنتی مسائل اور قد کی کمزوری بھی پیدا کرتے ہیں۔ ہم نے کم آمدنی اور درمیانی آمدنی والےممالک میں چھوٹے بچوں میں شیجیلا اسہال کے بوجھ، سیروٹائپس اور اینٹی بایوٹک مزاحمت کے نمونے معلوم کئے تاکہ ویکسین کے ٹرائل کی منصوبہ بندی اور آخرکار زیادہ بوجھ والے ممالک میں ویکسین کے تعارف کی رہنمائی کی جا سکے۔

طریقہ کار۔ گلوبل ہیلتھ کے لیے اینٹرکس (EFGH) مطالعہ ایک متوقع، سہولتی بنیاد پر ہائبرڈ نگرانی مطالعہ تھا جو 21 جون 2022 سے 25 اگست 2024 تک سات ممالک میں کیا گیا: کینیا، ملاوی، مالی، گیمبیا، بنگلہ دیش، پاکستان، اور پیرو۔ 6-35 ماہ عمر کے وہ بچے جو منتخب کردہ صحت کی دیکھ بھال کی سہولیات پر اچانک اسہال (گزشتہ 24 گھنٹے میں تین یا اس سے زیادہ غیر معمولی ڈھیلے یا پانی والے پاخانے جو 14 دن سے کم عرصے تک جاری رہیں) کے ساتھ پیش ہوئے، انہیں شامل کیا گیا۔ ہم نے دیکھ بھال کی تلاش کے مطابق ایڈجسٹ شدہ واقعہ کی تخمینے موجودہ آبادی کی گنتی سے اور صحت کی دیکھ بھال کے نمونے سے حاصل کیےجو 6-35 مہینے کے بچوں میں صحت کی سہولت کے دائرہ کار میں صحت کی دیکھ بھال کے استعمال کے سروے سے حاصل کیے گئے۔ ہم نے شیجیلا کو اس وقت منسوب کیا جب یہ مائیکروبایولوجیکل کلچر کے ذریعے یا مقداری پی سی آر(qPCR) کے ذریعے rectal swab سے ipaH مقداری سائیکل تھریش ہولڈ 5.29 یا اس سے کم کے استعمال سے پتہ چلا۔ ہم نے عام استعمال ہونے والی اینٹی بایوٹکس کے لئے ڈسک ڈفیوژن کے ذریعے اینٹی مائیکروبیل مزاحمت کا تعین کیا۔ ہم نے تمام شریک ممالک کی سائٹس اور دلچسپی کے اہم ذیلی گروپ(عمر، دست کی شدت، مطالعہ کا مہینہ شیجیلا انواع، اور سیروٹائپ) کے لئے ایڈجسٹ شدہ واقعہ کی تخمینے کی۔

نتائج۔ 9476 شامل شدہ بچوں میں سے4316 (5.٪45) لڑکیاں تھیں اور 5160 (5.٪54) لڑکے تھے، 881(3.٪9) میں کلچر کے ذریعے شیجیلا پایا گیا اور1870 (0.٪20) میں qPCR کے ذریعے(9354 بچوں میں qPCR کے نتائج دستیاب تھے)۔ شیجیلا فلیکسنیری غالب رہی (کلچر کے ذریعے 881 میں سے 497]2.٪56[اور qPCR کے ذریعے 1870 میں سے 756]4.٪39[، جس میں S flexneri 2a اور S flexneri 6 دونوں طریقوں سے سب سے عام سیروٹائپ تھیں۔ مطالعہ کے مختلف مقامات میں، کلچر کے ذریعے شیجیلاکی وجہ سے دست کی ایڈجسٹ شدہ شرح7.2 فی 100 بچوں-سال(95٪ CI 9.1 – 3.4)ملاوی میں اور 7.11 فی 100 بچوں-سال (3.8 – 2.24) پیرو میں پائی گئی، جبکہ qPCR کے ذریعے یہ شرح ملاوی میں 5.3 فی 100 بچوں-سال (5.2 – 4.5) اور گیمبیا میں 9 .26 فی 100 بچوں-سال (0.19 – 9.40) رہی۔ شیجیلا کے نمونے ڈسینٹری کے لیے WHO کی تجویز کردہ اینٹی بایوٹکس کے خلاف مزاحمت ظاہر کرتے ہیں، جو مقامات کے لحاظ سے مختلف ہے:سیپروفلوکساسین (2.37 ٪]دائرہ0.14 – 0.74[)، ایزیترومائیسن (1.22 ٪]2.1 – 2.34 [)، اور سیفٹراکسون(2.16 ٪]0.0 – 4.64 [)۔

تشریح۔ نوجوان بچوں میں LMICs میں شیجیلا کی وجہ سے دست عام ہیں، اور اس کی بڈھتی ہوئی اینٹی مائیکروبیل مزاحمت عالمی صحت کے لئے سنگین خطرہ ہے۔ سرکردہ چار گنا ویکسین کے امیدوار اس مطالعے میں شناخت شدہ شیجیلا کے زیادہ تر سیروٹائپس کا احاطہ کرتے ہیں۔ مجموعی طور پر، یہ ڈیٹا دونوں باتوں کی تصدیق کرتے ہیں کہ زیادہ بوجھ والے EFGH سائٹس میں شیجیلا ویکسینز کے سخت کلینیکل ٹرائلز کرانے کی ضرورت اور تیاری موجود ہے۔

**Research in context**

**Evidence before this study**

We searched PubMed, Scopus, Web of Science, and Embase from database inception to April 1, 2025, for publications in English, using the keywords “*Shigella*” “Shigellosis” “bacillary dysentery,” OR “Dysentery” AND “Africa” AND “South Asia”. Using these comprehensive search strategies, we identified studies that revealed a considerable burden of *Shigella* species (spp)-attributable diarrhoea in Africa and south Asia, with pooled prevalences of 5·9% (95% CI 4·9–7·0; adjusted 5·6% [4·7–6·6]) in Africa and 7% (6–7) in south Asia. PCR-based studies in south Asia report 15% (11–19) prevalence versus 6% (5–7) by culture, and African data mainly rely on culture, possibly underestimating prevalence. *Shigella flexneri* predominates (53 ·6% in Africa and 58% in south Asia) with *Shigella sonnei* at 11·5% in Africa and 19% in south Asia, *Shigella boydii* at 7·7% in Africa and 10% in south Asia, and *Shigella dysenteriae* at 10·1% in Africa and 9% in south Asia. Serotype 2a dominates within *S flexneri* strains. Data on antimicrobial resistance in *Shigella* isolates is scarce, particularly from among children living in Africa and Asia. Reported shigellosis incidence in the literature varied from 0·4 per 100 child-years to 43 cases per 100 child-years globally, contingent on factors such as diarrhoea severity and diagnostic methodology.

**Added value of this study**

This study identifies the dominant serotypes *S flexneri* and *S Sonnei* that contributed to more than 85% of *Shigella*-diarrhoea cases across seven countries. The study provides the most recent estimate of incidence rates of shigellosis in low-income and middle-income countries following the GEMS and MAL-ED studies. This study also provides estimates based on both culture and quantitative PCR, providing a more complete and policy-relevant estimate of disease burden by capturing both culture-confirmed cases and the broader attributable burden detected by molecular methods. Together, these complementary estimates inform vaccine trial endpoint selection, sample size planning, and realistic projections of vaccine-preventable and antimicrobial-resistant disease.

**Implications of all the available evidence**

This study provides up to date evidence on the burden of *Shigella* diarrhoea and resistance patterns of *Shigella* isolates to commonly used antibiotics among young children living in LMICs, the target population for *Shigella* vaccines. A multivalent *Shigella* vaccine, targeting the *S flexneri* and *S sonnei*, and providing early and sustained protection should be prioritised in light of the increasing resistance to WHO-recommended antibiotics. Vaccine trial planners and policy makers can use these data to optimise vaccine trial design and to aid in the prioritisation of an eventual *Shigella* vaccine.

## Introduction

Diarrhoeal diseases are among the leading causes of morbidity and mortality in children younger than 5 years, contributing to nearly 450 000 deaths globally each year, the majority of which occur in low-income and middle-income countries (LMICs).^1^, ^2^ *Shigella*, a Gram-negative bacillus, is a leading cause of dysentery and acute watery diarrhoea among children living in LMICs.^3^, ^4^ *Shigella*-attributed diarrhoea in children is associated with prolonged duration of diarrhoea, environmental enteric dysfunction, growth faltering, and stunting.^5^, ^6^, ^7^

The emergence of antimicrobial resistant *Shigella* infections in the past 10 years complicates clinical management. In south Asia, resistance to ciprofloxacin, the WHO-recommended first-line antibiotic, has been reported in more than 40% of *Shigella* isolates, and resistance to the alternative antibiotics (azithromycin and ceftriaxone) is on the rise.^8^, ^9^ Although, resistance to WHO-recommended antibiotics remains relatively low in sub-Saharan Africa, the trends seen in south Asia serve as a stark warning of the potential future global challenge in treating shigellosis.

Vaccines targeting *Shigella* complemented with water, sanitation, and hygiene preventive measures are needed to reduce the disease burden in LMICs. Several promising *Shigella* vaccines targeting children are in development. Phase 3 efficacy trials will be required in the target populations, thus necessitating well established clinical trial sites and updated baseline incidence rates for optimal sample size determination.^10^ The Enterics for Global Health (EFGH) *Shigella* surveillance study was a multicountry, health facility-based surveillance study that aimed to establish updated estimates of the incidence of *Shigella*-attributed medically attended diarrhoea and the prevalence of antimicrobial resistance among children aged 6–35 months in south Asia, sub-Saharan Africa, and South America. Data from this study could inform future vaccine trial design and policy makers considering vaccine introduction and show the infrastructure available to conduct large-scale *Shigella* vaccine trials.

## Methods

### Study design and participants

This prospective, facility-based, hybrid surveillance study recruited participants from June 21, 2022, to Aug 24, 2024, across seven countries in Africa (Kenya, Malawi, Mali, and The Gambia), south Asia (Bangladesh and Pakistan), and south America (Peru). Each country team enrolled up to 1400 participants over a 24-month period at health facilities (appendix 1 p 3). Children presenting with diarrhoea (defined as three or more abnormally loose or watery stools with or without blood in the previous 24 h) to study health facilities were screened for eligibility. Eligible children resided in the predefined catchment area, were experiencing a new, acute episode of diarrhoea (onset within the last 7 days after at least 2 diarrhoea-free days), and were accompanied by a consenting caregiver (appendix 1 p 4). There were no participant exclusion criteria.

Written informed consent was obtained from caregivers in the local language, following country-specific institutional review board requirements. The study protocol was reviewed and approved by respective institutional and ethical review boards (appendix 1 p 2). Detailed study methods have been previously published.^11^, ^12^, ^13^, ^14^, ^15^

### Procedures

Enrolled children underwent a physical examination by EFGH clinicians (trained on the study protocol and standard operating procedures to perform screening and enrolment), which included vital sign assessment (temperature, heart rate, and respiratory rate), dehydration assessment, and anthropometric measurements (length or height, weight, mid-upper arm circumference). Clinical history was abstracted from medical records when available and sociodemographic characteristics were collected through standardised caregiver interviews. Participant sex was sex-reported by the caregivers. Caregivers received a paper diary to record diarrhoea severity indicators, including diarrhoea duration, visible blood in stool, vomiting episodes, and medications received during the 14-day period following enrolment. Follow-up visits were conducted by trained research staff at 4 weeks (±7 days) and 3 months (±14 days) either at the participant's home or at the enrolling health facilities, and visits were considered missed if more than 30 days passed after the scheduled visit. Study clinicians were involved for unscheduled visits and in case of no recovery. If a child died, a mortality interview ascertaining conditions surrounding the death and timing of the death was conducted by research staff trained in ICD-10 at an appropriate time for the family.^16^ Assigned cause (or causes) of death were abstracted from medical records, the death certificate (if available), and an optional open-ended caregiver interview adapted from the 2016 WHO verbal autopsy instrument.^16^ Annually, a panel of EFGH Consortium clinicians and epidemiologists reviewed and assigned ICD-11-compliant causes of death through a consensus-based process.

Three rectal swabs (Pediatric FLOQswab, Copan Diagnostics, Murrieta, CA, USA) were collected from each child immediately after enrolment and before administration of medications by clinical staff. Participants were placed on their side or stomach and rectal swabs gently inserted and rotated 360 degrees three times before removal. One swab was placed in a dry sterile tube for eventual quantitative PCR (qPCR), and the other two were placed in separate transport media (Cary–Blair transport medium and semi-solid buffered glycerol saline) for standard microbiological culture and antibiotic susceptibility testing by disc diffusion (appendix 1 p 5). Whole stool was collected from children enrolled in Bangladesh and The Gambia to assess *Shigella* recovery from the two sample types and the results are available in a preprint paper.^17^

Details of the culture-based^13^ and molecular methods^14^ of pathogen detection are presented elsewhere. Pathogen-specific attributable quantification cycle (Cq) cut-offs from qPCR (appendix 1 p 6) were established based on whole stool Cq-specific odds ratios calculated using diarrhoea cases and controls matched to the EFGH age enrolment criteria from the Global Enterics Multicenter (GEMS) study and the Malnutrition and Consequences for Child Health and Development (MAL-ED) study. Only pathogens associated with diarrhoea in the GEMS or MAL-ED study were considered. To correct the cutoffs for use of rectal swabs versus whole stool, we calculated the mean difference in Cq values in paired samples from 2390 children with paired whole stool and rectal swab samples, both of which underwent qPCR testing using the same assay (appendix 1 p 6). A *Shigella*-attributed case of diarrhoea was defined as a child who had *Shigella* isolated by culture or *ipaH* detected by qPCR at or below the attributable Cq threshold of 29·5. For samples that were missing a qPCR rectal swab result but had a valid whole-stool result from the same participant in Bangladesh (n=28) and The Gambia (n=195), the whole-stool qPCR threshold (Cq cutoff) of 29·8 was used. Protocol deviations are specified in appendix 1 (p 7).

Population enumeration was conducted at each site to ascertain the population at risk in each facility's catchment area and to estimate the proportion of recent (within 14 days of the survey) enrolment-eligible patients with diarrhoea who sought care using a health-care utilisation survey. Catchment area populations of children aged 6–35 months were estimated contemporaneously with the 2-year enrolment period by surveying randomly selected clusters within catchment areas (appendix 1 p 8). We used WorldPop, an open access spatial demographic dataset, to divide the catchment areas into clusters by overlaying a grid, and then aggregating grid squares into clusters, with a goal cluster population of approximately 500. The health-care utilisation survey was conducted in all households within each cluster where at least one child aged 6–35 months resided.

Data collection was managed using Research Electronic Data Capture for diarrhoea case surveillance and on Android tablets using SurveyCTO (Dobility, Cambridge, MA, USA) for population enumeration.

### Statistical analysis

The detailed statistical analysis plan is available on ClinicalTrials.gov, NCT06047821. To estimate incidence of all *Shigella*-attributed medically attended diarrhoea in the catchment area, rather than the incidence in patients enrolled at EFGH facilities (ie, what was observed in the study), we incorporated three adjustment factors, (1) adjusting for enrolment to estimate incidence of *Shigella*-attributed medically attended diarrhoea in patients who sought care at EFGH facilities accounting for children who presented to the study facilities and were eligible but not enrolled (stratified by dysentery or watery diarrhoea); (2) adjusting for care seeking at EFGH facilities (*vs* other facilities) to estimate incidence among patients seeking care at any primary health-care facility in the catchment area; and (3) adjusting for any care seeking (*vs* no care seeking) with a propensity to seek care model (appendix 1 p 9) to estimate incidence among all children in the study catchment area.^18^ The propensity to seek care model estimated individual-level probabilities to seek care based on a set of clinical and sociodemographic characteristics hypothesised to be associated with care seeking, with model coefficients estimated using the health-care utilisation survey. The inverse of those probabilities was applied as individual-level weights such that patients least likely to seek care were weighted more than those most likely to seek care. Incidence was calculated for culture and qPCR for each EFGH country based on the estimated population of each catchment area, stratified by age, type of diarrhoea (dysentery or watery diarrhoea), whether or not the diarrhoea led to a hospitalisation (defined as an overnight stay), *Shigella* serogroup and serotype or subserotype, diarrhoea severity (GEMS,^3^ modified Vesikari score [MVS],^19^ *Shigella* mortality score,^20^ MVS with or without dysentery,^31^ MAL-ED,^22^ and Clark^23^), and study month to assess seasonality. Combined incidence was the average of country-specific incidences. Incidence was reported among potential *Shigella* vaccine targets assuming direct protection as well as assuming cross-protection (*S flexneri* 2a covering *S flexneri* 1a, 2b, 3b, 4a, and 5a, and *S flexneri* 3a covering *S flexneri* 2b, 3b, 4b, 5b, and X).^24^ Child-years at risk was estimated using the enumerated population of children (aged 6–35 months) while accounting for unenumerated households and clusters. All enrolled participants yielded a valid culture result and 122 (1·3%) children were missing qPCR results because of an invalid read. We applied an additional weight stratified by calendar month to account for missing qPCR test results. Bootstrapped 95% CIs were calculated for all incidence estimates. A sensitivity analysis calculated incidence using health-care seeking reported within 7 days (as opposed to 14 days as per the protocol) to assess any impact of recall bias.

Antimicrobial resistance was defined as a resistant or intermediate zone size to the corresponding antibiotic per Clinical and Laboratory Standards Institute guidelines as described elsewhere.^13^ Multidrug resistance was defined as resistance to three or more antibiotics. Extensively drug resistant was defined as resistance to all of the following: azithromycin, ciprofloxacin, ceftriaxone, trimethoprim-sulfamethoxazole, and ampicillin. Resistance to all WHO-recommend antibiotics was defined as resistance to azithromycin, ciprofloxacin, and ceftriaxone. All analyses were conducted in R (version 4.5.1).

### Role of the funding source

The funder of the study had no role in the study design, data collection, data analysis, data interpretation, or writing the report.

## Results

**Figure 1:**
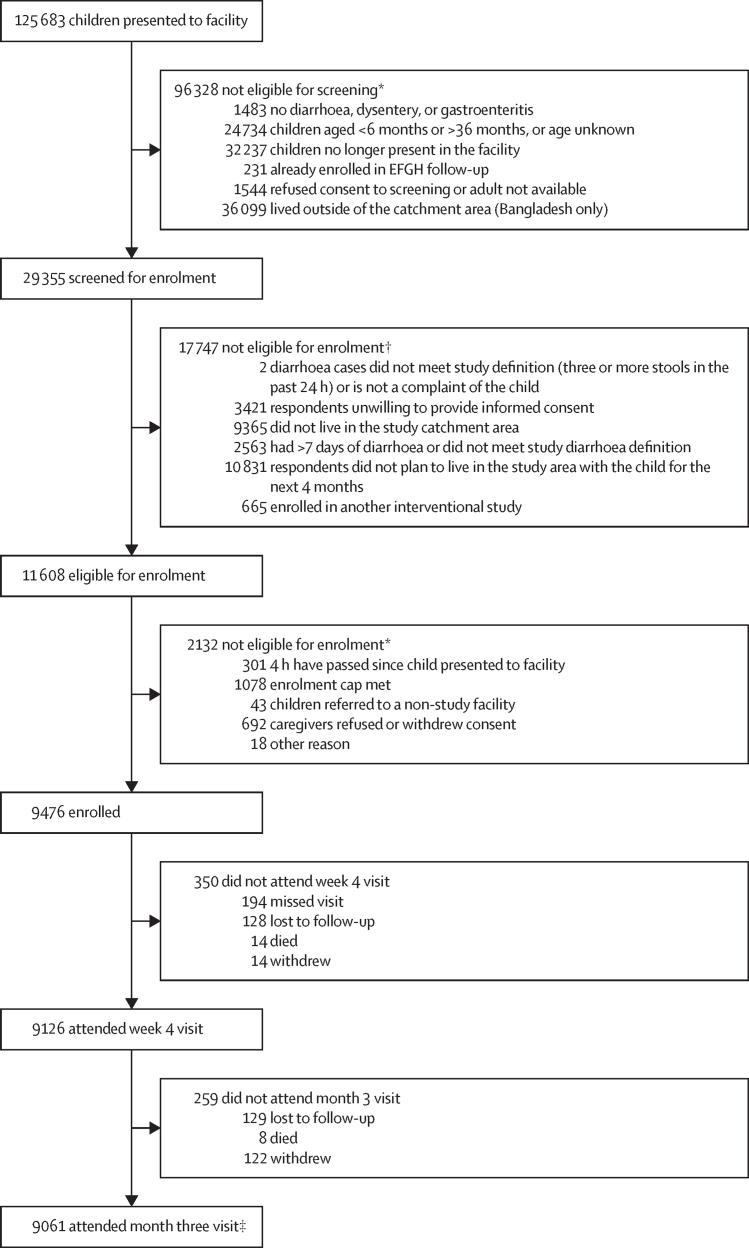
Diarrhoea case surveillance participant screening, enrolment and follow-up EFGH=The Enterics for Global Health Study. *Prescreening questions and questions assessing whether eligible children were enrolled were asked sequentially and therefore numbers for each indicator are not necessarily the total number children who presented to the facility and eligible children. †Participants could have had multiple reasons for ineligibility. ‡Included enrolled participants who attended the week 4 visit (n=9126) or missed the week 4 visit (n=194), but not participants who did not attend the week 4 visit due to death, withdrawal, or loss to follow-up.

Of 29 355 children screened, 9476 were enrolled in the study (figure 1). Overall, participant retention was high: 9126 (96·3%) of 9476 at week 4 and 9061 (97·2%) of 9320, excluding those who had died, were lost to follow-up, or withdrew at or before the week four visit, at month 3 across all seven sites (appendix 1 p 10). The median age of enrolled study participants was 14 months (IQR 9–21), and 4316 (45·5%) participants were female and 5160 (54·5%) were male (table). At enrolment, 2230 (23·5%) participants had some dehydration and 106 (1·1%) participants had severe dehydration. Additionally, 2234 (23·7%) children were stunted (length or height for age Z-score<–2), 1598 (16·9%) had wasting (weight for length or height Z-score<–2 or mid-upper arm circumference<11·5 cm), and 2056 (21·7%) were underweight (weight for age Z-score<–2).

**Table:** Demographic and clinical characteristics of participants enrolled in diarrhoeal case surveillance, by the Enterics for Global Health Study country site

		**Bangladesh (n=1361)**	**Kenya (n=1400)**	**Malawi (n=1399)**	**Mali (n=1400)**	**Pakistan (n=1400)**	**Peru (n=1117)**	**The Gambia (n=1399)**	**Total (n=9476)**
**Demographics**									
Sex									
	Female	596 (43·8%)	637 (45·5%)	651 (46·5%)	643 (45·9%)	655 (46·8%)	488 (43·7%)	646 (46·2%)	4316 (45·5%)
	Male	765 (56·2%)	763 (54·5%)	748 (53·5%)	757 (54·1%)	745 (53·2%)	629 (56·3%)	753 (53·8%)	5160 (54·5%)
Age, months									
	6–8	300 (22·0%)	330 (23·6%)	238 (17·0%)	330 (23·6%)	244 (17·4%)	189 (16·9%)	209 (14·9%)	1840 (19·4%)
	9–11	272 (20·0%)	230 (16·4%)	240 (17·2%)	270 (19·3%)	210 (15·0%)	176 (15·8%)	237 (16·9%)	1635 (17·3%)
	12–17	355 (26·1%)	359 (25·6%)	344 (24·6%)	365 (26·1%)	366 (26·1%)	347 (31·1%)	379 (27·1%)	2515 (26·5%)
	18–23	233 (17·1%)	236 (16·9%)	283 (20·2%)	248 (17·7%)	280 (20·0%)	238 (21·3%)	339 (24·2%)	1857 (19·6%)
	24–35	201 (14·8%)	245 (17·5%)	294 (21·0%)	187 (13·4%)	300 (21·4%)	167 (15·0%)	235 (16·8%)	1629 (17·2%)
Median age, months		13 (9–20)	14 (9–0)	15 (10–22)	13 (9–19)	15 (10–22)	14 (10–20)	16 (10–21)	14 (9–21)
Highest maternal education achieved									
	Less than primary school	283 (20·8%)	126 (9·0%)	105 (7·5%)	601 (42·9%)	501 (35·8%)	99 (8·9%)	539 (38·5%)	2254 (23·8%)
	Koranic school only	0	0	0	236 (16·9%)	34 (2·4%)	0	461 (33·0%)	731 (7·7%)
	Primary school or greater	1078 (79·2%)	1271 (90·8%)	1294 (92·5%)	551 (39·4%)	865 (61·8%)	1018 (91·1%)	397 (28·4%)	6474 (68·3%)
	Unknown or declined	0	3 (0·2%)	0	12 (0·9%)	0	0	2 (0·1%)	17 (0·2%)
Accompanying caregiver employment									
	Not employed	1269 (93·2%)	700 (50·0%)	814 (58·2%)	1279 (91·4%)	1313 (93·8%)	957 (85·7%)	804 (57·5%)	7136 (75·3%)
	Employed	73 (5·4%)	695 (49·6%)	575 (41·1%)	66 (4·7%)	78 (5·6%)	150 (13·4%)	564 (40·3%)	2201 (23·2%)
	Unknown or other	19 (1·4%)	5 (0·4%)	10 (0·7%)	55 (3·9%)	9 (0·6%)	10 (0·9%)	31 (2·2%)	139 (1·5%)
Children aged <5 years in household		1 (1–1)	1 (1–2)	1 (1–2)	3 (2–5)	2 (1–2)	1 (1–2)	4 (3–7)	2 (1–3)
Months exclusively breastfed		6 (5–6)	6 (5–6)	6 (4–6)	6 (1–6)	6 (4–6)	6 (5–6)	5 (2–6)	6 (4–6)
Wealth index		4 (3–5)	4 (3–4)	3 (2–3)	3 (2–3)	2 (1–3)	2 (1–2)	2 (2–3)	3 (2–4)
**Water, sanitation, and hygiene characteristics**									
Drinking water source*									
	Improved source	1359 (99·9%)	950 (67·9%)	1392 (99·5%)	1400 (100·0%)	1397 (99·8%)	1017 (91·0%)	1382 (98·8%)	8897 (93·9%)
	Unimproved source	2 (0·1%)	450 (32·1%)	7 (0·5%)	0	3 (0·2%)	100 (9·0%)	17 (1·2%)	579 (6·1%)
Sanitation access†									
	Improved source	1357 (99·7%)	842 (60·1%)	1057 (75·6%)	1362 (97·3%)	1333 (95·2%)	752 (67·3%)	1074 (76·8%)	7777 (82·1%)
	Unimproved source	4 (0·3%)	558 (39·9%)	342 (24·4%)	38 (2·7%)	67 (4·8%)	365 (32·7%)	325 (23·2%)	1699 (17·9%)
**Clinical characteristics**									
Dysentery‡		206 (15·1%)	148 (10·6%)	138 (9·9%)	93 (6·6%)	234 (16·7%)	170 (15·2%)	241 (17·2%)	1230 (13·0%)
Dehydration§									
	None	1191 (87·5%)	656 (46·9%)	1348 (96·4%)	1236 (88·3%)	1185 (84·6%)	214 (19·2%)	1310 (93·6%)	7140 (75·3%)
	Some	168 (12·3%)	685 (48·9%)	49 (3·5%)	154 (11·0%)	207 (14·8%)	897 (80·3%)	70 (5·0%)	2230 (23·5%)
	Severe	2 (0·1%)	59 (4·2%)	2 (0·1%)	10 (0·7%)	8 (0·6%)	6 (0·5%)	19 (1·4%)	106 (1·1%)
Received antibiotic before care seeking, per caregiver report		237 (17·4%)	33 (2·4%)	54 (3·9%)	53 (3·8%)	77 (5·5%)	60 (5·4%)	42 (3·0%)	556 (5·9%)
Received age-appropriate rotavirus vaccine, per country guidelines¶		NA	814 (67·2%)	919 (96·3%)	1168 (95.0%)	845 (85·3%)	858 (90·7%)	1364 (98·3%)	5968 (88.8%)
Care seeking before enrolment visit									
	None	487 (35·8%)	1286 (91·9%)	1153 (82·4%)	1102 (78·7%)	1227 (87·6%)	878 (78·6%)	1314 (93·9%)	7447 (78·6%)
	Inpatient or outpatient health facility	445 (32·7%)	23 (1·6%)	69 (4·9%)	2 (0·1%)	121 (8·6%)	98 (8·8%)	31 (2·2%)	789 (8·3%)
	Other care seeking	429 (31·5%)	91 (6·5%)	177 (12·7%)	296 (21·1%)	52 (3·7%)	141 (12·6%)	54 (3·9%)	1240 (13·1%)
Hospitalised‖		112 (8·2%)	58 (4·1%)	1 (0·1%)	1 (0·1%)	9 (0·6%)	8 (0·7%)	54 (3·9%)	243 (2·6%)
Duration of diarrhoea before care seeking, days		3 (2–4)	1 (1–2)	2 (1–2)	2 (1–2)	2 (1–3)	3 (1–4)	2 (1–2)	2 (1–3)
MVS**		8 (7–12)	9 (6–11)	7 (5–9)	6 (5–9)	8 (6–10)	9 (7–11)	7 (6–10)	8 (6–10)
	Mild	692 (50·8%)	668 (47·7%)	946 (67·6%)	1046 (74·7%)	883 (63·1%)	456 (40·8%)	888 (63·5%)	5579 (58·9%)
	Moderate	175 (12·9%)	289 (20·6%)	235 (16·8%)	173 (12·4%)	239 (17·1%)	294 (26·3%)	248 (17·7%)	1653 (17·4%)
	Severe	494 (36·3%)	443 (31·6%)	218 (15·6%)	181 (12·9%)	278 (19·9%)	367 (32·9%)	263 (18·8%)	2244 (23·7%)
Moderate or severe diarrhoea by MVS or dysentery††		796 (58·5%)	802 (57·3%)	538 (38·5%)	407 (29·1%)	644 (46·0%)	722 (64·6%)	673 (48·1%)	4582 (48·4%)
Global Enterics Multicenter Study‡‡									
	Less-severe diarrhoea	966 (71·0%)	584 (41·7%)	1214 (86·8%)	1161 (82·9%)	989 (70·6%)	184 (16·5%)	1062 (75·9%)	6160 (65·0%)
	Moderate-to-severe diarrhoea	395 (29·0%)	816 (58·3%)	185 (13·2%)	239 (17·1%)	411 (29·4%)	933 (83·5%)	337 (24·1%)	3316 (35·0%)
*Shigella* mortality score§§		2 (0–3)	4 (0–4)	0 (0–0)	0 (0–2)	2 (0–2)	4 (4–6)	0 (0–0)	0 (0–4)
	Mild	1221 (89·7%)	1174 (83·9%)	1393 (99·6%)	1343 (95·9%)	1273 (90·9%)	585 (52·4%)	1351 (96·6%)	8340 (88·0%)
	Moderate	74 (5·4%)	163 (11·6%)	5 (0·4%)	55 (3·9%)	122 (8·7%)	521 (46·6%)	20 (1·4%)	960 (10·1%)
	Severe	66 (4·8%)	63 (4·5%)	1 (0·1%)	2 (0·1%)	5 (0·4%)	11 (1·0%)	28 (2·0%)	176 (1·9%)
Clark score¶¶		8 (6–11)	7 (5–9)	5 (4–7)	4 (3–6)	6 (4–9)	6 (4–8)	6 (5–8)	6 (4–8)
	Mild	770 (56·6%)	971 (69·4%)	1176 (84·1%)	1264 (90·3%)	1033 (73·8%)	853 (76·4%)	1077 (77·0%)	7144 (75·4%)
	Moderate to severe	591 (43·4%)	429 (30·6%)	223 (15·9%)	136 (9·7%)	367 (26·2%)	264 (23·6%)	322 (23·0%)	2332 (24·6%)
Malnutrition and Consequences for Child Health and Development Study score‖‖		6 (5–9)	6 (5–8)	5 (4–6)	4 (3–6)	6 (4–7)	7 (5–8)	5 (4–7)	6 (4–7)
	Non-severe	493 (36·2%)	511 (36·5%)	877 (62·7%)	973 (69·5%)	666 (47·6%)	328 (29·4%)	713 (51·0%)	4561 (48·1%)
	Severe	868 (63·8%)	889 (63·5%)	522 (37·3%)	427 (30·5%)	734 (52·4%)	789 (70·6%)	686 (49·0%)	4915 (51·9%)
**Anthropometry**									
Stunting***									
	Not stunted	1029 (75·7%)	1139 (81·6%)	988 (71·1%)	1217 (86·9%)	844 (61·0%)	868 (78·8%)	1106 (79·3%)	7191 (76·3%)
	Stunted	331 (24·3%)	257 (18·4%)	402 (28·9%)	183 (13·1%)	540 (39·0%)	233 (21·2%)	288 (20·7%)	2234 (23·7%)
Wasting†††									
	None	1119 (82·3%)	1337 (95·7%)	1327 (95·4%)	1086 (77·6%)	913 (65·8%)	1052 (95.5%)	1000 (71·7%)	7834 (83·1%)
	Moderate	206 (15·1%)	48 (3·4%)	53 (3·8%)	261 (18·6%)	334 (24·1%)	44 (4·0%)	307 (22·0%)	1253 (13·3%)
	Severe	35 (2·6%)	12 (0·9%)	11 (0·8%)	53 (3·8%)	141 (10·2%)	6 (0·5%)	87 (6·2%)	345 (3·7%)
Underweight‡‡‡									
	None	1081 (79·4%)	1297 (92·6%)	1224 (87·5%)	1088 (77·7%)	799 (57·2%)	1008 (90·2%)	919 (65·7%)	7416 (78·3%)
	Moderate	222 (16·3%)	82 (5·9%)	139 (9·9%)	239 (17·1%)	370 (26·5%)	90 (8·1%)	351 (25·1%)	1493 (15·8%)
	Severe	58 (4·3%)	21 (1·5%)	36 (2·6%)	73 (5·2%)	227 (16·3%)	19 (1·7%)	129 (9·2%)	563 (5·9%)

Data are n (%) or median (IQR). HAZ=height for age Z-score. JMP=WHO–UNICEF Joint Monitoring Programme for Water Supply, Sanitation, and Hygiene. LAZ=length for age Z-score. MUAC=mid-upper arm circumference. MVS=modified Vesikari score. NA=not applicable. WAZ=weight for age Z-score. WLZ=weight for length Z-score.

* Drinking water source was classified according to the JMP as unimproved (water from a river, dam, lake, pond, stream, canal or irrigation channel, and unprotected wells or springs) or improved (water piped into the dwelling, yard or plot, public taps or standpipes, tube wells or boreholes, protected dug wells or springs, rainwater collection, water from a tanker-truck, water from a cart with a small tank or drum, filtered or unfiltered water kiosks, bottled water, or sachet water.

† Sanitation access was classified according to the JMP as unimproved (no facility, bush or field, flush toilets to elsewhere, pit latrines without slab floor, bucket toilets, or hanging toilets or latrines), or improved (flush toilets to piped sewer systems, septic tanks or unknown location, ventilated improved pit latrines, pit latrines with slab floors, or composting toilets).

‡ Blood in the stool as reported by caregiver during the diarrhoeal episode or by clinical diagnosis.

§ Based on WHO criteria. Severe dehydration is at least two of the following signs: lethargy, abnormally sunken eyes, drinks poorly, and skin pinch >2 s. Some dehydration is at least two of the following signs: restless or irritable, abnormally sunken eyes, drinks eagerly, and skin pinch 1–2 s.

¶ Two (Kenya and Mali) or three (Malawi, Pakistan Peru, and The Gambia) documented doses by age 6 months. Rotavirus vaccine is not part of the national immunisation schedule for Bangladesh. Do not sum to column totals due to missing data.

‖ Defined as an overnight stay (child was on the ward from at least 12 am to 6 am or self-discharged before that timeframe.

** Defined as in PATH Vesikari Clinical Severity Scoring System Manual.^19^ Duration of diarrhoea: 1–4 days (1 point), 5 days (2 points), and ≥6 days (3 points). Maximum number of stools in 24 h period: 1–3 (1 point), 4–5 (2 points), and ≥6 (3 points). Duration of vomiting: 1 day (1 point), 2 days (2 points), and ≥3 days (3 points). Maximum number of vomiting episodes in 24 h period: 1 (1 point), 2–4 (2 points), and ≥5 (3 points). Axillary temperature 36·6–37·9°C (1 point), 38·0–38·4°C (2 points), and ≥38·5°C (3 points). Dehydration: 1–5% (2 points) and ≥6% (3 points). Treatment: rehydration (1 point) and hospitalisation (2 points).

†† Defined as by Pavlinac and colleagues^21^ as a MVS of 9+ or presence of visible blood in stool.

‡‡ Defined as by Kotloff and colleagues.^3^ Moderate-to-severe diarrhoea is defined as presenting to a health facility with diarrhoea and severe or some dehydration (by WHO criteria), visible blood in stool, or inpatient admission. Less-severe diarrhoea is defined as presenting to a health facility without moderate-to-severe diarrhoea.

§§ Defined as by Pavlinac and colleagues.^20^ Duration of diarrhoea through day of presentation: 1–3 days (0 points), 4–5 days (2 points), and ≥6 days (3 points). WHO-defined dehydration categories: severe (8 points), some (4 points), and none (0 points). Inpatient admission (5 points).

¶¶ Defined as by Clark and colleagues.^23^ Duration of diarrhoea: 1–4 days (1 point), 5–7 days (2 points), and >7 days (3 points). Maximum number of stools in 24 h period: 2–4 (1 point), 5–7 (2 points), and >7 (3 points). Duration of vomiting: 2 days (1 point), 3–5 days (2 points), and >5 days (3 points). Maximum number of vomiting episodes in 24 h period: 1–3 (1 point), 4–6 (2 points), and >6 (3 points). Duration of reported fever: 1–2 days (1 point), 3–4 days (2 points), and ≥5 days (3 points). Rectal temperature 38·0–38·2°C (1 point), 38·3–38·7°C (2 points), and ≥38·8°C (3 points). Behavioural signs: irritable or less playful (1 point), lethargic or listless (2 points), and seizures (3 points).

‖‖ Defined as by Lee and colleagues.^22^ Duration of diarrhoea: 2–4 days (1 point), 5–7 days (2 points), and ≥8 days (3 points). Maximum number of stools in 24 h period: <5 loose stools per 24 h (1 point), 5–7 loose stools per 24 h (2 points), and ≥8 stools per 24 h (3 points). Duration of vomiting: 1 day (1 point), 2 days (2 points), and ≥3 days (3 points). Duration of reported fever: ≥1 days (1 point). Confirmed temperature: ≥37·5°C (confirmed by field worker; 2 points). Dehydration: some (2 points) and severe (3 points).

*** Severe stunting: HAZ or LAZ less than −3. Moderate stunting: HAZ or LAZ −3 to −2. Does not sum to total because 51 participants were missing HAZ or LAZ data.

††† Severe wasting: WLZ less than −3 or MUAC less than 11·5 cm. Moderate wasting: WLZ −3 to −2 or MUAC 11·5 to 12·5 cm. Does not sum to total because six participants were missing WLZ or MUAC data.

‡‡‡ Severely underweight: WAZ less than −3. Moderately underweight: WAZ −3 to −2. Does not sum to total because four participants were missing WAZ data.

Among the 54 609 children aged 6–35 months visited during the population enumeration (country range 3023 to 17 127), 4853 (8·9%) had diarrhoea in the previous 2 weeks by caregiver report. Of those caregivers of children who had diarrhoea in the past 2 weeks and who consented to participate in the health-care utilisation survey (n=4794), caregivers reported that 1731 (36·1%) sought care at an inpatient or outpatient hospital or health centre (including EFGH facilities) in the previous 2 weeks, ranging from 84 (15·9%) of 528 in Mali to 707 (55·1%) of 1283 in Pakistan (appendix 1 p 21). The adjusted incidence rate of diarrhoea was 75·2 per 100 child-years and ranged from 37·1 in Malawi to 135·3 in Peru (appendix 1 p 11).

**Figure 2:**
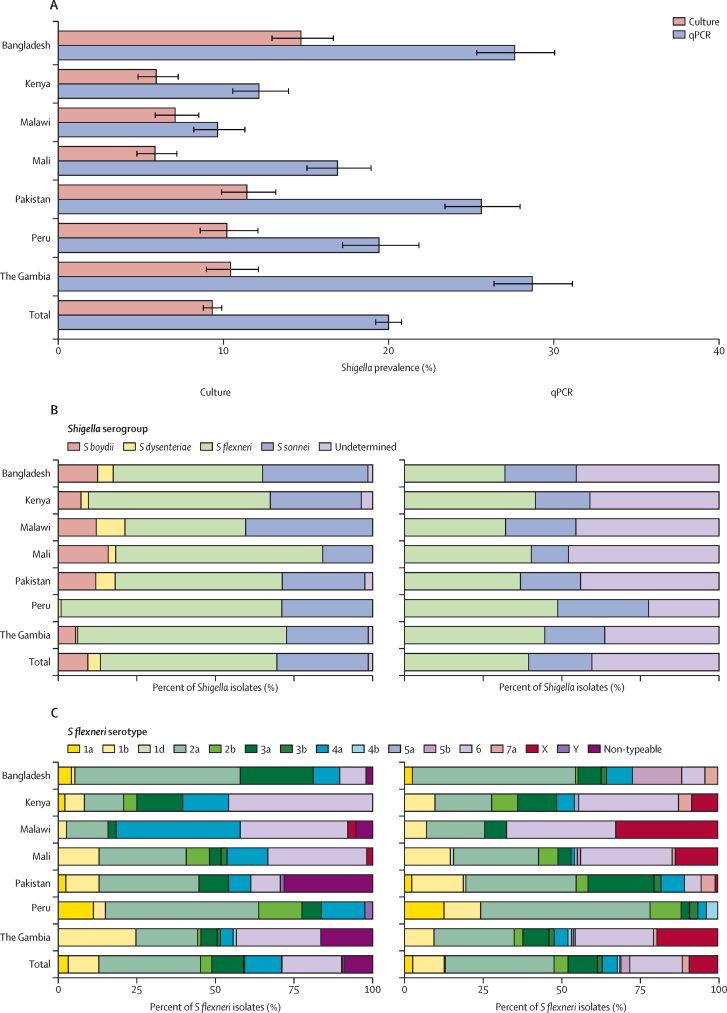
*Shigella* prevalence and serotype distribution by EFGH country site and laboratory method (A) The prevalence of *Shigella* was defined as the percentage of samples positive for culture or below the attributable quantification cycle threshold for qPCR with uncertainty expressed as Wilson binomial with 95% CIs. The serogroup distribution (B) and *S flexneri* serotype distribution (C) are the number and percent of isolates testing positive for a particular serogroup and (among *S flexneri* isolates) for a particular serotype. Totals do not sum to total participants testing positive for *Shigella* because a small number of participants (three for culture [884 isolates from 881 participants] and 47 for qPCR [1917 isolates from 1870 participants]) had multiple serotypes or subserotypes. *S flexneri* 7a was not assessed by culture and *S dysenteriae* and *S boydii* were not assessed by qPCR and are thus classified as undetermined in B. Site-specific serotype and serogroup distributions are in appendix 1 (p 16). EFGH=The Enterics for Global Health Study. qPCR=quantitative PCR. *S boydii*=*Shigella boydii. S dysenteriae*=*Shigella dysenteriae. S flexneri*=*Shigella flexneri. S sonnei*=*Shigella sonnei*.

**Figure 3:**
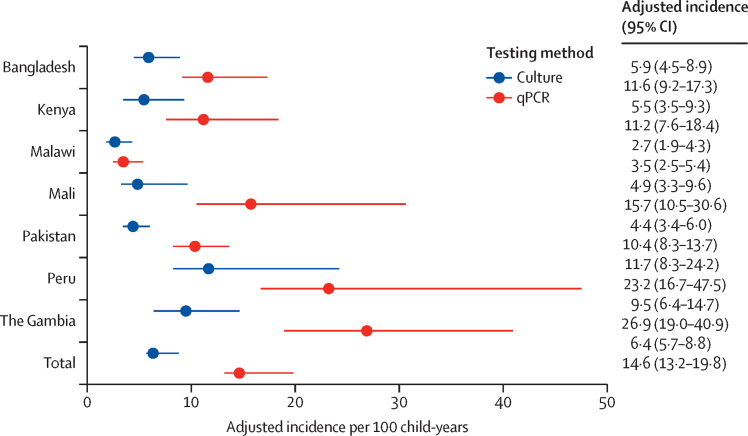
Adjusted *Shigella* incidence of medically attended diarrhoea by EFGH country site and laboratory method Incidence was defined as the estimated number of *Shigella* medically attended diarrhoea cases per 100 child-years. Incidence was computed by dividing the number of enrolled participants who tested positive for *Shigella* by culture or who were below the attributable quantification cycle threshold by qPCR by the estimated child-years at risk among the population of children aged 6 months to 35 months residing in catchment area adjusting incidence for children who were not enrolled and therefore not tested for *Shigella* but were eligible as well as for children in the catchment area who reported diarrhoea of similar severity to facility-enrolled cases but did not report seeking care at an EFGH facility. Totals are the average of country-level estimates, and 95% CIs were generated using bootstrapping. EFGH=The Enterics for Global Health Study. qPCR=quantitative PCR.

*Shigella* culture prevalence was 9·3% with 884 unique isolates among 881 participants (figure 2). Among 9354 children with qPCR results, *Shigella*-attributable qPCR prevalence was 20·0% with 1917 unique detections at attributable levels from 1870 enrolled participants (989 [52·9%] of which did not have *Shigella* isolated by culture; appendix 1 p 12). The adjusted incidence of *Shigella*-attributed diarrhoea by culture ranged from 2·7 per 100 child-years (95% CI 1·9–4·3) in Malawi to 11·7 per 100 child-years (8·3–24·2) in Peru, with a mean incidence of 6·4 per 100 child-years (5·7–8·8; figure 3). The use of qPCR yielded notably higher adjusted incidence rates, ranging from 3·5 per 100 child-years (2·5–5·4) in Malawi to 26·9 per 100 child-years (19·0–40·9) in The Gambia, with a mean rate of 14·7 per 100 child-years (13·2–19·8). Crude incidence rates and semi-adjusted incidence rates are in appendix 1 (p 13).

**Figure 4:**
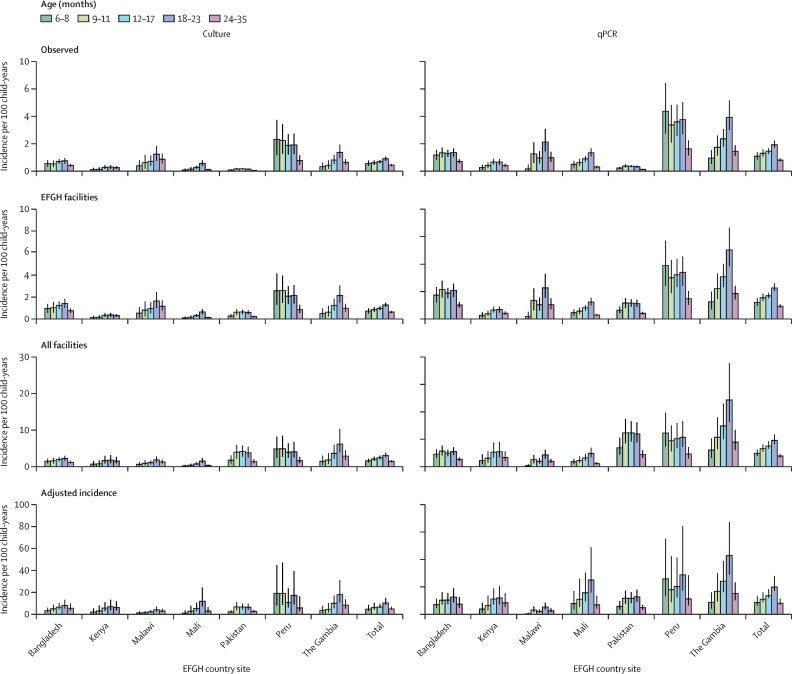
Age-stratified *Shigella*-attributed medically attended diarrhoea incidence by EFGH country site and laboratory method Incidence was defined as the number of observed medically attended diarrhoea cases per 100 child-years. Incidence was stratified and computed for each age group of interest by dividing the number of enrolled participants of a specific age who tested positive for *Shigella* for culture or who were below the attributable quantification cycle threshold by qPCR by the estimated child-years at risk among children of a corresponding age residing in catchment area. Total incidences are the mean of country-level incidences. 95% CIs were generated using bootstrapping and details of incidence calculations are in appendix 1 (p 9). EFGH=The Enterics for Global Health Study. qPCR=quantitative PCR.

*Shigella-*attributed diarrhoea incidence peaked in children aged 18–24 months but exceeded 4·5 per 100 child-years by culture and 10·0 per 100 child-years by qPCR, in all age groups, including children aged 6–9 months (figure 4). Mild *Shigella-*attributed diarrhoea was more common than the more severe forms by all severity definitions (appendix 1 p 14). We did not observe major seasonal trends in *Shigella* incidence other than possible peaks in Malawi around November and December and in Mali and The Gambia between June and August of each year (appendix 1 p 15).

The distribution of *Shigella* species across EFGH sites varied (figure 2; appendix 1 p 16), with 38 (38·4%) of 99 *S flexneri* isolates in Malawi to 80 (70·2%) of 114 *S flexneri* isolates in Peru and 13 (15·9%) of 82 *S sonnei* isolates in Mali and 40 of 99 (40·4%) *S sonnei* isolates in Malawi. *S dysenteriae* comprised 35 (4·0%) of 885 of *Shigella* isolates but no cases of *S dysenteriae* type 1 were observed*.* Among the 497 *S flexneri* isolates, the most common serotypes and subserotypes were *S flexneri* 2a (n=161, 32·4%), *S flexneri* 6 (n=94, 18·9%), *S flexneri* 4a (n=58, 11·7%), and *S flexneri* 3a (n=50, 10·1%). However, there was variation by EFGH country site, with *S flexneri* 2a being the leading *S flexneri* subserotype in three sites (Bangladesh, Pakistan, and Peru); *S flexneri* 6 the leading subserotype in Kenya, Mali, and The Gambia; and *S flexneri* 4a being the leading subserotype in Malawi. The distribution of serotypes and sub-serotypes of *Shigella* by qPCR were similar (appendix 1 p 16) but the absolute number of children with *S flexneri*-attributed diarrhoea was higher by qPCR (n=756) than by culture (n=497). Assuming no cross-protection across *S flexneri* sub-serotypes, the leading quadrivalent vaccine candidates (S *flexneri* 2a, 3a, 6, and *S sonnei;* and *S flexneri* 1b, 2a, 3a, and *S sonnei*) cover 562 (63·6%) and 516 (58·4%) of the 884 culture-proven *Shigella* cases and 846 (44·1%) and 795 (41·5%) of the 1917 cases diagnosed by qPCR, respectively. Assuming cross-protection with shared O-antigen structure, these vaccine candidates cover 659 (74·5%) and 613 (69·3%) of the 884 cases confirmed by culture, and 1047 (54·6%) and 996 (52·0%) of the 1917 cases confirmed by qPCR, respectively. The incidence rates of *Shigella*-attributed diarrhoea by serogroups, serotypes, and in the leading vaccine candidates are in appendix 1 (p 14).

**Figure 5:**
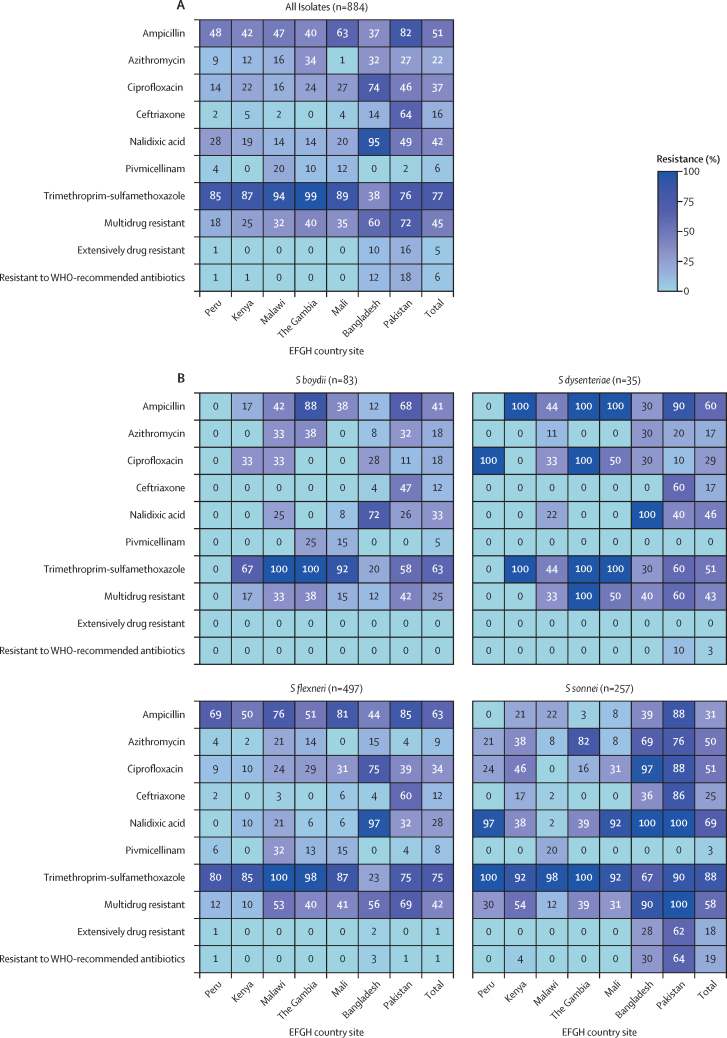
Antimicrobial resistance among *Shigella* isolates overall and by *Shigella* serogroup The percentage of *Shigella* culture-positive isolates resistant to antibiotics or combined indicators of interest was determined overall, by EFGH country site, and stratified by *Shigella* serogroup. Resistance was defined as non-susceptibility (resistant or intermediate zone size classifications) per CLSI guidelines. Multidrug resistance was defined as resistance to three or more antibiotics. Extensively drug resistant was defined as resistance to all of the following: azithromycin, ciprofloxacin, ceftriaxone, trimethoprim-sulfamethoxazole, and ampicillin. Resistance to all WHO-recommended antibiotics was defined as resistance to azithromycin, ciprofloxacin, and ceftriaxone. Samples that were resistant, intermediate, or susceptible by CLSI guidelines are shown in appendix 1 (p 17). CLSI=Clinical and Laboratory Standards Institute. EFGH=The Enterics for Global Health Study. *S boydii*=*Shigella boydii. S dysenteriae*=*Shigella dysenteriae. S flexneri*=*Shigella flexneri. S sonnei*=*Shigella sonnei*.

Resistance to trimethoprim-sulfamethoxazole (n=667, 76·6% [country range 38·5–98·6%]), ampicillin (n=454, 51·4% [country range 37·0–82·5%]), nalidixic acid (n=368, 41·6% [country range 14·1–95·0%]), and ciprofloxacin (n=329, 37·2% [country range 14·0–74·0%]) was common among the 884 cultured *Shigella* isolates (figure 5; appendix 1 p 17). Moderate resistance levels were observed to azithromycin (n=195, 22·1% [country range 1·2–34·2%]) and ceftriaxone (n=143, 16·2% [country range 0·0–64·4%]) and resistance to pivmecillinam was lower (n=53, 6·0% [country range 0·0–20·2%]). Overall, resistance to all WHO-recommended treatments was evident (n=54, 6·1%), most commonly in Pakistan (29 [18·1%] of 160) and Bangladesh (23 [11·5%] of 200). All serogroups had a similar susceptibility pattern to pivmicellinam; however, susceptibility to ampicillin, azithromycin, ciprofloxacin, and nalidixic acid varied among serogroups. *S sonnei* had lower levels of resistance to ampicillin (79 [30·7%] of 257) compared with *S flexneri* (315 [63·8%] of 497).

Among the 9476 children enrolled in the diarrhoea case surveillance, 22 (0·2%) deaths were recorded during the 3-month follow-up period, including five (0·2%) among 1931 participants with *Shigella* by culture or attributed by qPCR (appendix 1 p 18). The median age at death was 11·5 months (IQR 8·5–16·8), with a median time of 21·5 days (10·0–31·0) from enrolment to death. A cause of death was determined for 12 of the 22 known deaths, with the leading causes identified as sepsis (three [25·0%] of 12), pneumonia (two [16·7%] of 12), and hypovolemic shock (two [16·7%] of 12).

Among the 1870 children with qPCR-attributed *Shigella* and 878 children with culture-positive *Shigella*, 582 (31·1%) and 201 (22·9%), respectively, had another pathogen identified by qPCR at an attributable quantity (appendix 1 p 19). The prevalence (by qPCR) of co-aetiologies differed by site, with co-aetiology present in 136 (16·8%) of 214 patients with *Shigella*-attributed medically attended diarrhoea in Peru but 180 (48·3%) of 373 patients with *Shigella*-attributed medically attended diarrhoea in Bangladesh. Overall, the most frequent co-pathogens by qPCR (n=1870, with some variation in denominators due to pathogen-specific invalid results) were heat-stable enterotoxigenic *Escherichia coli* (236 [12·8%] of 1839), rotavirus (136 [7·4%] of 1841), and *Cryptosporidium* (90 [4·8%] of 1857). There was variability of the top three co-aetiologies by country, with adenovirus replacing heat-stable enterotoxigenic *E coli* in Peru and *Campylobacter* replacing *Cryptosporidium* in Bangladesh. The likelihood of co-aetiology did not differ when stratifying by diarrhoea severity.

## Discussion

In this seven-country *Shigella* surveillance study, we confirmed *Shigella* to be an important cause of diarrhoea and dysentery in young children. The alarmingly high frequency of antibiotic resistance to guideline-recommended therapy, particularly in south Asia, underscores the need for *Shigella* vaccines for young children in LMICs. The leading quadrivalent vaccine candidates would target between 40% and 75% of *Shigella* cases, and although estimated protection was slightly lower with qPCR than culture, the absolute number of cases averted more than doubled by qPCR compared with culture due to its higher diagnostic sensitivity. The non-negligible burden of *Shigella-*attributed diarrhoea in children aged 6–12 months and peak in the 18–24-month age range highlights early vaccination is necessary to avert the majority of *Shigella* cases in young children.

The discrepancy between culture and qPCR-based detection of *Shigella* is well recognised.^25^, ^26^ Stool culture, although highly specific, has limited sensitivity because *Shigella* is a fastidious organism that loses viability rapidly outside the host and is easily overgrown by commensal flora during transport or delayed processing and prior antibiotic use. In contrast, molecular assays such as qPCR detect *Shigella* DNA directly from stool, capable of identifying low bacterial loads and, possibly, non-viable organisms.^25^, ^27^

Our findings that *S flexneri* followed by *S sonnei* were the most common serogroups at all sites and across the two diagnostic methods reaffirms GEMS^24^ culture-based and MAL-ED^28^ qPCR-based results. There was some variation in the most prevalent *S flexneri* subserotypes by country sites and diagnostic method; however, subserotypes 2a and 6 were consistently highest. Variation in the *S flexneri* subserotype relative importance by culture and qPCR might reflect true genotypic or phenotypic discordance,^29^ a proclivity for one subserogroup to be culture-negative and thus more detected in qPCR-positive *Shigella* cases, or error in laboratory assignment, such as ambiguous visual assignment with antisera. Ongoing qPCR-testing and sequencing of discordant isolates will be reported elsewhere and will help clarify the true serotype.

Adjusted incidence of *Shigella* was lowest among infants aged 6–8 months with a gradual increase until a peak in the 18–23-month-old group. *Shigella*-attributed diarrhoea incidence peaking in the second year of life is consistent with other studies including MAL-ED, which documented the median time of first *Shigella* infection to be 14 months and 30% of children experiencing at least one episode of *Shigella*-attributed diarrhoea in their first 2 years.^5^, ^25^ Although lowest in the 6–8 month age group, the adjusted incidence of *Shigella*-attributed diarrhoea was non-negligible and the incidence rates in children aged 9–11 month exceeded those in children aged 24–35 months. Given that *Shigella-*attributed diarrhoea severity is highest in the youngest children as is the risk of death and growth faltering,^20^, ^30^ a *Shigella* vaccine achieving early and sustained protection is likely to have the greatest impact.

There was a concerningly high level of resistance to WHO-recommended antibiotics. Of the available oral antibiotic options we tested, *Shigella* isolates were most susceptible to pivmecillinam. This extended spectrum penicillin is the recommended second-line therapy for *Shigella* at the International Centre for Diarrhoeal Disease, Bangladesh, but to our knowledge, pivmecillinam is not widely used because of its challenging dosing regimen (15 mg/kg every 6 h for 5 days) and availability only in tablet form (200 mg per tablet). *S sonnei* isolates appeared to be more resistant to antibiotics than *S flexneri* isolates, consistent with other studies,^31^ particularly in Bangladesh and Pakistan, but *S flexneri* had higher levels of ampicillin resistance compared with *S sonnei*.

The variability in identification of a second enteric pathogen at attributable quantities among *Shigella-*attributed diarrhoea cases is consistent with other studies.^5^, ^25^ The lower proportion of co-aetiology among cultured *Shigella* might suggest that culture identifies children with viable *Shigella;* hence, clearer *Shigella-*specific attribution of the diarrhoea. Because a *Shigella* vaccine is expected to have *Shigella*-specific efficacy, the question of whether other possible co-aetiologies need to be excluded from primary endpoint definitions has been raised. Rotavirus trials did not exclude co-pathogens in primary endpoint definitions despite similarly high co-pathogens and secondary analyses excluding these pathogens had modest impacts on efficacy.^32^

Diarrhoea case fatality was lower than observed in other facility-based diarrhoea studies: GEMS (1·6%)^3^ and Antibiotics for Children with Severe Diarrhoea (0·7% in placebo group and 0·5% in azithromycin group).^33^ This lower case-fatality is consistent with marked reductions in overall diarrhoeal disease mortality during the past decade.^34^ The EFGH study supported the treatment of diarrhoea in accordance with WHO-recommended care guidelines, which might have impacted mortality rates.

By employing a hybrid surveillance approach, this study was able to efficiently estimate total community incidence of *Shigella*-attributed medically attended diarrhoea, requiring fewer resources and less time than would be needed in a cohort study. Despite our extensive efforts to capture a representative sample, our study's facility-based design might have introduced selection bias, because it might not fully reflect the incidence of *Shigella*-attributed diarrhoea in the general population, particularly among children whose caregivers did not seek medical care or sought care after working hours. Our adjusted incidence rates accounted for the fact that not all eligible children were enrolled and we improved on the adjustment for health-care seeking behaviours by using a propensity to seek care model enabling a nuanced weighting of children seeking care for diarrhea that accounted for differing care seeking behaviours by clinical and sociodemographic characteristics. We believe adjusted incidence rates represent the most accurate depiction of incidence rates in an eventual individually-randomised vaccine trial, as such trials will include close follow-up and care-seeking encouragement at specific health facilities. The health-care utilisation survey relied on caregiver report of diarrhoea and signs and symptoms of severity but might have been limited by recall bias. However, the sensitivity analysis that limited to 7-day recall instead of 14-day recall did not result in meaningfully different incidence rates (appendix 1 p 20). Conducting health-care utilisation surveys and population enumeration in parallel with recruitment of patients with diarrhoea captured seasonal differences in care seeking and household sizes. However, the study's 24-month duration, although comprehensive, might not capture long-term seasonal trends or year-to-year variations in Shigella incidence. We were unable to confidently attribute diarrhoea to *Shigella* when other pathogens were present at an attributable level, nor were we able to attribute *Shigella* to mortalities. Verbal autopsies and mortality attribution are a known challenge in low-resourced settings, and although we utilised best practices for cause of death assignment, including consensus-based decision-making among ICD-10 trained clinical experts, the causes of death assignment might be imperfect. Finally, due to budget limitations we were unable to determine minimum-inhibitory concentrations (MICs) or genotypic resistance in *Shigella* isolates, which can better inform resistance trends over time, emerging resistance patterns, and subtle MIC shifts that precede clinical failures.

*Shigella* remains an important cause of diarrhoea in young children living in LMICs and its high antibiotic resistance requires urgent intervention. Leading quadrivalent *Shigella* vaccine candidates are estimated to target the majority of *Shigella* moderate-or-severe diarrhoea and should be prioritised. EFGH catchment areas exhibit a high burden of *Shigella* disease in young children and the strong research infrastructure positions EFGH sites to implement robust, methodolically rigorous *Shigella* vaccine trials.

**This online publication has been corrected. The corrected version first appeared at thelancet.com/lancetgh on March 24, 2026**

### EFGH Consortium

### Contributors

### Equitable partnership declaration

### Data sharing

The EFGH statistical analysis plan (https://clinicaltrials.gov/study/NCT06047821) and study protocol (https://academic.oup.com/ofid/issue/11/Supplement_1) were made publicly available. The de-identified and anonymised dataset is published online (https://search.vivli.org/doiLanding/studies/PR00011860/isLanding) and analytic code is available at https://github.com/efghdata/Yousafzai-et-al.-2026-Lancet-Global-Health.

## Declaration of interests

We declare no competing interests.

## Supplementary Materials

Supplementary appendix 1

Equitable Partnership Declaration
