# Correction to *Lancet Glob Health* 2026; published online March 11 https://doi.org/10.1016/S2214-109X(25)00534-0

**DOI:** 10.1016/S2214-109X(26)00092-6

**Published:** 2026-03-24

*Yousafzai MT, Cornick J, Penataro Yori P, et al. The incidence and antimicrobial resistance of Shigella-attributable diarrhoea in young children in low-income and middle-income countries from the multicountry Enterics for Global Health (EFGH) Shigella Surveillance Study: a prospective, facility-based hybrid surveillance study.* Lancet Glob Health *2026; published online March 11 https://doi.org/10.1016/S2214-109X(25)00534-0*—In the colour key for figure 2C of this Article, the fourth label from the left should have read “2a”. In figure 5, from left to right, the x-axes labels should have read “Peru”, “Kenya”, “Malawi”, “The Gambia”, “Mali”, “Bangladesh”, “Pakistan”, and “Total”. This correction has been made as of March 24, 2026.

