# Supplementary material for: The incidence and antimicrobial resistance of Shigella-attributable diarrhoea in young children in low-income and middle-income countries from the multicountry Enterics for Global Health (EFGH) Shigella Surveillance Study: a prospective, facility-based hybrid surveillance study
Source: Lancet Glob Health. 2026 Mar 11;14(5):e749–61. doi: 10.1016/S2214-109X(25)00534-0 (PMC13106044; doi:10.1016/S2214-109X(25)00534-0)
Supplement: Supplementary appendix 1 [file mmc1.pdf]

THE LANCET
Global Health

Supplementary appendix 1

This appendix formed part of the original submission and has been peer reviewed. We post it as supplied by the authors.

Supplement to: Yousafzai MT, Cornick J, Penataro Yori P, et al. The incidence and antimicrobial resistance of *Shigella*-attributable diarrhoea in young children in low-income and middle-income countries from the multicountry Enterics for Global Health (EFGH) *Shigella* Surveillance Study: a prospective, facility-based hybrid surveillance study. *Lancet Glob Health* 2026; published online March 11. https://doi.org/10.1016/S2214-109X(25)00534-0.

Supplementary Materials

Contents

EFGH Study Teams Collaborators 1
EFGH Study Institutional Review Boards 2
Table S1: Clinical facilities participating in Diarrhea Case Surveillance. 3
Table S2: Inclusion Criteria for Medically-Attended Diarrhea Cases 4
Table S3: Stool Specimen Processing 5
Table S4. qPCR attributable cycle-threshold cutoffs for rectal swabs and whole stool samples. 6
EFGH Study Protocol Deviations 7
Table S5. Estimation of the catchment areas' population sizes. 8
Table S6. Incidence Calculation Definitions 9
Figure S1. Diarrhea surveillance participant screening, enrollment and follow-up by EFGH country site. 10
Table S7. Incidence of all-cause diarrhea, by EFGH country. 11
Table S8. Comparison of *Shigella* culture positivity and attribution by qPCR among enrollment participants. 12
Table S9. *Shigella* incidence by culture and qPCR, by EFGH country 13
Table S10. *Shigella* diarrhea severity-stratified adjusted incidence of MAD by culture and qPCR, by EFGH country 14
Figure S2. Seasonality of adjusted *Shigella* MAD incidence by EFGH country site and laboratory method. 15
Table S11. Site-specific *Shigella* species and serotype distribution by culture and qPCR. 16
Figure S3. Country-specific antimicrobial resistance to culture-confirmed *Shigella* isolates. 17
Table S12. ICD-11 causes of death for all known mortality events occurring within the EFGH three-month follow-up period. 18
Figure S4. Prevalence of qPCR-attributable enteric co-pathogens among children 6-35 months with qPCR-attributable or culture positive *Shigella* MAD, by site and diarrhea severity. 19
Table S13. *Shigella* incidence using a healthcare-seeking adjustment among diarrhea reported within the past seven days. 20
Table S14. Characteristics of the population enumeration and healthcare utilization survey, by study site. 21

EFGH Study Teams Collaborators

Isaiah Akello, Ethel Alumando, Manase Amolloh, Raphael Anyango, Md Taufiqur Rahman Bhuiyan, Bubacarr E. Ceesay, Umberto D'Alessandro, Ryan Dodd, Sarah E. Elwood, Irum Fatima, Erika Feutz, Md Ismail Hossen, Mahzabeen Ireen, Samba Juma Jallow, Sheikh Jarju, Mehrab Karim, Youssouf Keita, Zubair Latif, Clement Lefu, Anya M. Lewin, Rebecca Maguire, Katia Manzanares Villanueva, Christine J. McGrath, Arianna Rubin Means, Chloe Morozoff, Stephen Munga, Vitumbiko Yagontha Munthali, Maureen Ndalama, Latif Ndeketa, Caren Oreso, Tackeshy Pinedo Vasquez, Firdausi Qadri, Syed Qudrat-E-Khuda, Lucero Romaina-Cachique, Queen Saidi, Doh Sanogo, Olivia Lang Schultes, Wagner Valentino Shapiama Lopez, Kirkby D. Tickell, Moussa Traore, Loyda Fiorella Zegarra Paredes

EFGH Study Institutional Review Boards

This study was conducted according to Good Clinical Practice (GCP), including Good Clinical Laboratory Practice (GCLP), the Declaration of Helsinki, IRB and local rules and regulations specific to each EFGH country. This protocol was subject to ethical approval from the Institutional Review Boards (IRBs) at each EFGH site.

Bangladesh:
The Institutional Review Board of International Centre for Diarrhoeal Disease Research, Bangladesh gave ethical approval for this work [Approval #: PR-21114]

Kenya:
The KEMRI Scientific and Ethics Review Unit of the Kenya Medical Research Institute gave ethical approval for this work [Approval #: PROTOCOL NO. KEMRI/SERU/CGHR/403/4362]. Kenya Medical Research Institute, has been licensed by The National Commission for Science, Technology and Innovation to conduct research as per the provision of the Science, Technology and Innovation Act, 2013 (Rev.2014) in Siaya on the topic: ENTERICS FOR GLOBAL HEALTH: SHIGELLA SURVEILLANCE STUDY (EFGH) for the period ending : 27/January/2024 [License No: NACOSTI/P/22/15379 & NACOSTI/P/23/23350].

Malawi:
The College of Medicine Research Ethics Committee of Kamuzu University of Health Sciences gave ethical approval for this work [Approval #'s: P.10/21/3437]. The Central University Research Ethics Committee D of the University of Liverpool gave ethical approval for this work [Approval #: 10596].

Mali:
Universite Des Sciences, Des Techniques et des Technologies de Bamako gave ethical approval for this work [Approval #'s: 00000918, 00000964, 0000091, 00000189, 00000440, 0000022, 00000383, 00000279]. The University of Maryland, Baltimore Institutional Review Board gave ethical approval for this work [Approval #'s: HP-00098210, HM-HP-00098210-1, HM-HP-00098210-2, HM-HP-00098210-3].

Pakistan:
The Ethics Review Committee of The Aga Khan University gave ethical approval for this work [Approval #'s: 2021-6932-19680, 2022-6932-21888, 2022-6932-23332, 2022-6932-23399, 2023-6932-24228, 2023-6932-25760]. The Pakistan National Institutes of Health, Health Research Institute, National Bioethics Committee gave ethical approval for this work [Approval #'s: No.4-87/NBC-746/22/1556 , No.4-87/NBC-746/22/160, No.4-87/NBC-746-Exten/23/1577].

Peru:
Comité Institucional de Ética en Investigación Prisma gave ethical approval for this work [Approval #'s: CE0043.22, CE0669.23, CE0674.22, CE0513.23, CE0669.23]

The Gambia:
The Observational / Interventions Research Ethics Committee of the London School of Hygiene & Tropical Medicine gave ethical approval for this work [Approval #: 26515].

U.S. Coordination:
The Human Subjects Divisions of The University of Washington, Seattle, USA, The University of Virginia, Charlottesville, USA, and Emory University, Atlanta, USA, determined that the activities of the coordinating bodies do not constitute human subjects research as defined by federal regulations. Therefore, review and approval by these IRBs was not required.

Table S1: Clinical facilities participating in Diarrhea Case Surveillance.

Facility	Facility Type	Hours of recruitment	First day of enrollment	Last day of enrollment
**Bangladesh**				
Mugda Medical College Hospital	Public hospital (tertiary-level)	Sunday – Thursday 8:30am –5:00pm	July 4, 2022	June 20, 2024
icddr,b Dhaka Hospital	Public hospital (tertiary-level)		June 21, 2022	June 20, 2024
EFGH Field Office	Public treatment center (primary-level)		June 21, 2022	June 20, 2024
**Kenya**				
Siaya County Referral Hospital	Public-Level 4	Monday – Thursday 8:00am – 5:00pm, Friday 8:00am – 4:00pm	August 1, 2022	July 31, 2024
Lwak Mission Hospital	Private (Mission)-Level 3			
Ongielo Health Center	Public-Level 3			
Akala Health Center	Public-Level 3			
Ndienya Health Center	Public-Level 3			
Bar Agulu Health Center	Public-Level 2			
**Malawi**				
Ndirande Health Center	Public, health center	Monday – Friday 8:00am – 5:00pm	August 3, 2022	August 2, 2024
**Mali**				
Banconi CSCOM (Asacoba)	Public, peripheral	Monday – Friday 8:00am – 4:00pm	August 16, 2022	August 15, 2024
Asacodjeneka CSCOM	Public, peripheral			
Asacodjip CSCOM	Public, peripheral			
CSREF Commune 1	Public, district			
**Pakistan**				
Sindh Government Hospital, Karongi	Public, district	Monday – Saturday 9:00am – 3:00pm	September 26, 2022	August 24, 2024
Sindh Government Hospital, Ibrahim Hyderi	Public, district		October 15, 2022	August 24, 2024
Khidmat e Alam Medical Center	Charity, peripheral		August 25, 2022	August 24, 2024
Abbasi Shaheed Hospital	Public, tertiary		September 5, 2022	August 24, 2024
Bhains Colony VPT Center	Private/free, peripheral		August 30, 2022	August 24, 2024
Ali Akbar Shah VPT Center	Private/free, peripheral		August 25, 2022	August 24, 2024
**Peru**				
San Juan de Miraflores	Public, primary care	Monday – Saturday 8:00am – 4:00pm	August 9, 2022	August 8, 2024
Progreso de San Juan Bautista	Public, primary care			
America de San Juan Bautista	Public, primary care			
Santo Tomas de San Juan Bautista	Public, primary care			
Modelo	Public, primary care			
**The Gambia**				
Basse Hospital	Public, Secondary level	Monday – Thursday 8:00am – 4:30pm, Friday 8:00am – 12:30pm	August 16, 2022	August 15, 2024
Gambisara Health Center	Public, primary level			

Table S2: Inclusion Criteria for Medically-Attended Diarrhea Cases

1.	Child is six to 35 months of age.
2.	Child resides within the pre-defined study catchment area at least last 14 days.
3.	Primary caregiver and child plan to remain at their current residence for at least next four months.
4.	Primary caregiver is able to provide informed consent (legal age or emancipated minor) and provides consent within a common language for which translations are available.
5.	Child presents to health facility with diarrhea (three or more abnormally loose or watery stools in the previous 24 hours) with or without the presence of blood.
6.	Child presented during working hours or less than four hours has passed since the child presented before working hours to a health facility.
7.	Child presented with acute (onset within seven days of study enrollment) and represent a new (onset after at least two diarrhea-free days) diarrhea episode.
8.	Caregiver is willing to have child participate in follow-up visits at week four and month three.
9.	Willingness to have samples collected from the child (rectal swabs and stool samples collection at enrollment).
10.	Site enrollment cap has not been met.
11.	Child is not being referred to a non-EFGH facility at the time of screening.

Table S3: Stool Specimen Processing

Method Component	Description
Primary Plating	Plated on MacConkey and XLD agar and incubated aerobically for 24 hours at 37°C.
Colony Testing	Up to ten colonies with characteristic appearance were tube-tested using the following:<br>- Motility Indole Ornithine (MIO)<br>- Triple Sugar Iron (TSI)<br>- Lysine Decarboxylase Test (LDC)<br>- Urea Agar
Serotyping	Isolates consistent with *Shigella* biochemically were serotyped using group-specific polyvalent antisera (Denka Seiken, MAST Assure, Hardy Diagnostics). Additional monovalent testing was performed for Shigella flexneri isolates as per manufacturer's instructions (Horne et al., 2024).
Antimicrobial Susceptibility Testing	Confirmed *Shigella* isolates were tested for antimicrobial susceptibility using the disk diffusion method, with interpretation based on the Clinical and Laboratory Standards Institute (CLSI, 2022) guidelines.
Quality Control	All laboratories underwent proficiency testing before beginning specimen processing and continued proficiency tests every six months throughout the study. Additionally, all TAC (TaqMan Array Card) qPCR run files were reviewed centrally by assay developers at the University of Virginia.

Table S4. qPCR attributable cycle-threshold cutoffs for rectal swabs and whole stool samples.

	Attributable Ct cutoff			
	Whole stool samples			Rectal swab samples
**Pathogen**	**GEMS**	**MAL-ED**	**EFGH***	**EFGH†**
Adenovirus 40/41	24·7	22·3	23·5	24·2
Aeromonas	21·2	22·0	21·6	21·9
Astrovirus	23·8	24·9	24·4	26·2
Campylobacter jejuni/coli	-‡	19·9	19·9	19·6
Cryptosporidium	26·9	23·7	25·3	25·8
Cyclospora cayetanensis	31·9	-‡	31·9	32·7
Entamoeba histolytica	31·3	29·8	30·6	31·4
Cystoisospora belli	-‡	32·4	32·4	33·2
Norovirus GII	20·6	27·7	24·2	26·0
Rotavirus	32·3	31·3	31·8	33·6
Salmonella	31·3	-‡	31·3	31·4
Sapovirus	17·1	26·8	21·9	23·9
Shigella	29·4	30·1	29·8	29·5
ST-ETEC	23·0	25·2	24·2	25·6
tEPEC	-‡	17·4	17·4	18·6
Vibrio cholerae	33·4	30·3	31·9	32·7

ABCD: Antibitoics for Childhood Diarrhea Study, Ct: cycle threshold, EFGH: Enterics for Global Health Study, GEMS: Global Enterics Multicenter Study, MAL-ED: Malnutrition and Enteric Disease Study, ST-ETEC: heat-stable enterotoxigenic *E. coli*, tEPEC: typical enteropathogenic *E. coli*.

* Calculated as the mean of GEMS and MAL-ED whole stool Ct value cutoffs.

† Calculated using the EFGH whole stool Ct value cutoffs and subtracting the pathogen-specific rectal swab conversion calculated as the mean difference from ABCD and EFGH (includes 2390 pairs [433 from ABCD and 1957 from EFGH]).

‡ No associated with diarrhea in this study, only one study was used to determine EFGH Ct in this case.

EFGH Study Protocol Deviations

The following minor protocol deviations occurred during the course of the study:

- In the Gambia, 228 enrolled children were missing a rectal swab qPCR result (227 due to contamination and one due to an internal control failure). Because whole stool was also collected in the Gambia, as part of the laboratory methods optimization sub-study, we used the whole stool qPCR results (accounting for the sample type) for those with whole stool qPCR results available (195/228). In Bangladesh, where whole stool was also collected for the laboratory sub-study, there were 39 internal control failures from rectal swab samples that were able to be replaced with whole stool qPCR results for 28.
- All country sites were meant to work up rectal swabs transported in Cary-Blair and mBGS media to enable the eventual comparison of these two media types. However, in Peru, samples from the first 281 enrolled children were only worked up via one media type (Cary-Blair) and not both. This deviation was corrected and the remaining participants' samples were worked up from both transport media.

Table S5. Estimation of the catchment areas' population sizes.

Country	Children 6-35 months enumerated (A): n	Households enumerated (B): %*	Clusters enumerated: n	Study area enumerated (C): %†	Estimated children 6-35 months (D): n‡
Bangladesh	17 127	98·0	731	100·0	17 584
Kenya	4601	99·1	157	27·4	16 973
Malawi	4258	92·5	94	80·0	5948
Mali	5921	99·4	348	34·0	17 506
Pakistan	10 024	97·9	893	13·7	74 459
Peru	3023	81·3	236	98·9	3742
The Gambia	9655	99·9	726	100·0	9669
**Total**	**54 609**	**95·4**	**3185**	**65·9**	**145 882**

* The percentage of households approached that were reached within three visits and who provided verbal consent. This adjustment was computed at the study cluster level and indicators are the site cluster mean.

† The percentage of the overall study area (in square meters) that has been enumerated (area completed clusters/total study area).

Table S6. Incidence Calculation Definitions.

Definition	Description
Incidence of *Shigella* medically-attended diarrhea (MAD) enrolled at EFGH facilities	Observed *Shigella* MAD cases at EFGH facilities divided by the child-years at risk in the EFGH clinic catchment area (see "Population at risk").
Incidence of *Shigella* MAD who sought care at EFGH facilities	Estimated *Shigella* MAD cases at EFGH facilities accounting for children eligible for enrollment but not enrolled (see "Enrollment adjustment") divided by the child-years at risk in the EFGH clinic catchment area (see "Population at risk").
Incidence of *Shigella* MAD who sought care in the catchment area	Estimated *Shigella* MAD cases at all facilities in the catchment area accounting for children eligible for enrollment but not enrolled (see "Enrollment adjustment") divided by the child-years at risk in the EFGH clinic catchment area (see "Population at risk"). Includes a weight to account for care seeking that occurred at other facilities in the catchment area (see "Non-EFGH care facility adjustment").
Incidence of *Shigella* MAD in the community (**primary outcome - referred to as "Adjusted incidence" in the manuscript**)	Estimated *Shigella* MAD cases in the catchment area accounting for children eligible for enrollment but not enrolled (see "Enrollment adjustment") and care seeking (see "Healthcare seeking adjustment" and "Non-EFGH facility adjustment") divided by the child-years at risk in the EFGH clinic catchment area (see "Population at risk").
Population at risk	The total estimated number of children six to 35 months of residing in the catchment area was estimated using a population enumeration. Catchment areas were divided into clusters and visited in random order over the period of enrollment to visit all catchment area households and enumerate children six to 35 months of age. The estimated child-years at risk was computed by taking the estimated number of children enumerated in the catchment area accounting for households and study clusters that were not reached divided by the enrollment period of Diarrhea Case Surveillance (DCS).
Enrollment adjustment	Accounts for children eligible for enrollment but not enrolled due to reasons including lack of consent, limited staff or clinic capacity, timing of presentation, relocation plans, involvement in other studies, referral to non-EFGH facilities, enrollment caps, and cases where >4 hours had passed since presentation to screening.
Non-EFGH care facility adjustment	Weights were computed for each EFGH country site accounting for children who sought care in the catchment area but at a non-EFGH facility and therefore could not be observed in DCS.
Healthcare seeking adjustment	A propensity score model was used to adjust incidences to account for children in the community of a similar diarrhea syndrome whose caretakers did not seek care. Variables included EFGH country site, age, sex, days with fever, days with vomiting, maximum vomiting episodes in one day, maximum stools in one day, blood in stool, and wealth quintile. Propensity score weights are computed at the individual level for each child enrolled.
Missing sample result adjustment	An additional weight was used for all incidences to account for missing sample results among enrolled DCS participants. This adjustment was computed by calendar month to account for secular trends in missing sample result and *Shigella* seasonality and is computed by taking the inverse of the percent of available sample results for each calendar month. As culture results were available for all enrolled participants, this was only used to account for missing qPCR results.

DCS: diarrhea case surveillance, MAD: medically attended diarrhea.

Figure S1. Diarrhea surveillance participant screening, enrollment and follow-up by EFGH country site.

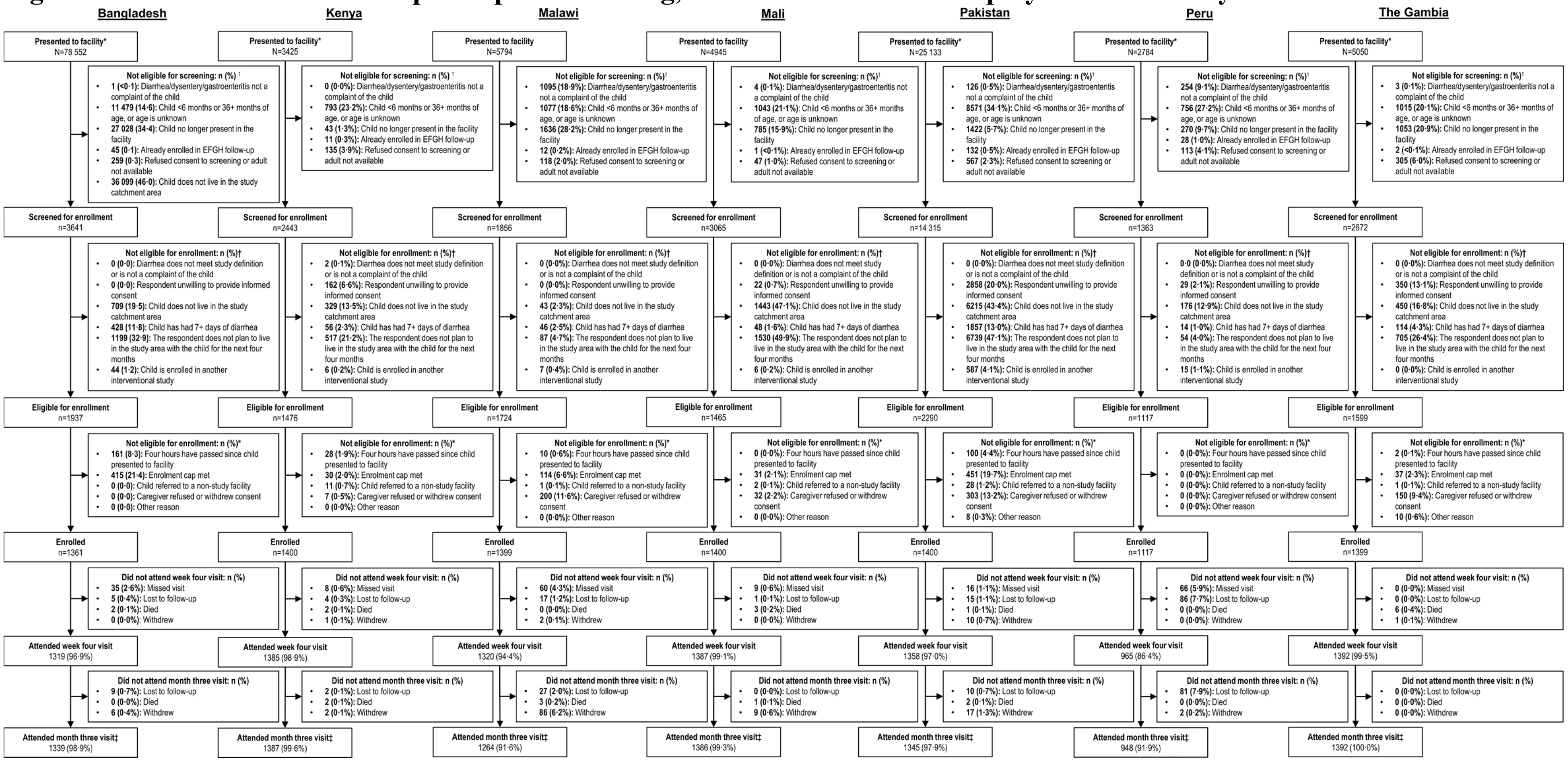

* Prescreening questions and questions assessing whether eligible children were enrolled were asked sequentially and therefore frequencies and percentages for each indicator are not necessarily among total children who presented to the facility (for reasons children were not screened) and eligible children (for children who were eligible but not enrolled).

† Reasons participants did not meet eligibility do not sum to total as participants could have screened out for multiple reasons.

‡ Among enrolled participants who attended the week four visit or missed the week four visit but not due to death, withdrawal or lost to follow-up (LTFU).

Table S7. Incidence of all-cause diarrhea, by EFGH country.

	Enrolled medically-attended diarrhea (MAD) cases: n			Diarrhea incidence per 100 child-years (95% CI)*			
**Country**	Watery diarrhea	Dysentery	Total	Enrolled at EFGH facilities (observed)	Estimated cases at seeking care EFGH facilities	Estimated cases seeking care in the catchment area	Estimated cases in the catchment area (adjusted incidence)
Bangladesh	1192	169	1361	3·9	8·0	12·6	41·8
Kenya	1308	92	1400	4·1	5·2	24·9	84·8
Malawi	1305	94	1399	11·8	15·8	17·9	37·1
Mali	1331	69	1400	4·0	4·6	10·8	87·9
Pakistan	1294	106	1400	1·0	4·1	26·1	43·9
Peru	976	141	1117	14·9	17·0	31·9	135·3
The Gambia	1184	215	1399	7·2	12·2	35·3	95·5
**Total‡**	**8590**	**886**	**9476**	**6·7**	**9·6**	**22·8**	**75·2**

MAD: medically-attended diarrhea

* Incidence is calculated at the facility-level and summed to get country-level incidence and is the number of confirmed watery diarrhea cases per 100 child-years plus the number of confirmed dysentery cases per 100 child-years at risk. Observed incidence is not adjusted, estimated incidence at EFGH facilities adjusts incidence by accounting for children who were not enrolled and therefore not tested for but were eligible, estimating cases in the catchment area seeking care additionally adjusts incidence for the percentage who sought care at EFGH facilities among all care seeking, and the adjusted incidence is additionally adjusted for children in the catchment area who reported diarrhea of similar severity to facility-enrolled cases but did not report seeking care. 95% confidence intervals were generated using bootstrapping. Details of incidence rate calculations are shown in Table S6.

‡ Totals are the average of country-level estimates.

Table S8. Comparison of *Shigella* culture positivity and attribution by qPCR among enrollment participants.

**qPCR**	Negative	**Culture** Positive	Total
Not attributable	7398	86	7484
Attributable*	1087	783	1870
No qPCR result available	110	12	122
**Total**	**8595**	**881**	**9476**

* Defined as an ipaH cycle threshold (Ct) below the attributable cutoff.

Table S9. *Shigella* incidence by culture and qPCR, by EFGH country.

Country	Confirmed *Shigella* cases: n Watery diarrhea	Dysentery	Total	*Shigella* MAD incidence per 100 child-years (95% CI)* Enrolled at EFGH facilities (observed)	Estimated cases at seeking care at EFGH facilities	Estimated cases seeking care in the catchment area	Estimated cases in the catchment area (adjusted incidence)
**Confirmed by culture†**							
Bangladesh	129	71	200	0·6 (0·5, 0·7)	1·0 (0·9, 1·2)	1·6 (1·3, 2·0)	5·9 (4·5, 8·9)
Kenya	64	18	82	0·2 (0·2, 0·3)	0·3 (0·2, 0·4)	1·4 (0·9, 2·3)	5·5 (3·5, 9·3)
Malawi	85	14	99	0·8 (0·6, 1·1)	1·1 (0·8, 1·5)	1·3 (0·9, 1·7)	2·7 (1·9, 4·3)
Mali	64	17	81	0·2 (0·2, 0·3)	0·3 (0·2, 0·3)	0·6 (0·5, 0·9)	4·9 (3·3, 9·6)
Pakistan	124	35	159	0·1 (0·1, 0·1)	0·4 (0·3, 0·5)	2·6 (2·1, 3·4)	4·4 (3·4, 6·0)
Peru	85	29	114	1·5 (1·2, 1·9)	1·7 (1·4, 2·2)	3·2 (2·4, 4·9)	11·7 (8·3, 24·2)
The Gambia	73	73	146	0·8 (0·6, 1·0)	1·2 (0·9, 1·5)	3·4 (2·4, 5·2)	9·5 (6·4, 14·7)
**Total‡**	**624**	**257**	**881**	**0·6 (0·5, 0·7)**	**0·9 (0·8, 1·0)**	**2·0 (1·8, 2·4)**	**6·4 (5·7, 8·8)**
**Attributable by molecular diagnostics (qPCR)§**							
Bangladesh	266	107	373	1·1 (0·9, 1·2)	2·0 (1·8, 2·2)	3·1 (2·7, 3·8)	11·6 (9·2, 17·3)
Kenya	141	29	170	0·5 (0·4, 0·6)	0·6 (0·5, 0·8)	3·0 (2·1, 4·7)	11·2 (7·6, 18·4)
Malawi	113	18	131	1·1 (0·9, 1·5)	1·5 (1·2, 2·0)	1·7 (1·3, 2·3)	3·5 (2·5, 5·4)
Mali	207	29	236	0·7 (0·6, 0·8)	0·8 (0·7, 0·9)	1·8 (1·4, 2·6)	15·7 (10·5, 30·6)
Pakistan	297	57	354	0·2 (0·2, 0·3)	1·0 (0·9, 1·1)	6·3 (5·1, 7·8)	10·4 (8·3, 13·7)
Peru	163	51	214	2·9 (2·4, 3·5)	3·3 (2·7, 3·9)	6·1 (4·6, 8·8)	23·2 (16·7, 47·5)
The Gambia	262	130	392	2·1 (1·7, 2·6)	3·3 (2·7, 4·2)	9·6 (6·7, 14·7)	26·9 (19·0, 40·9)
**Total‡**	**1449**	**421**	**1870**	**1·2 (1·1, 1·4)**	**1·8 (1·7, 2·0)**	**4·5 (4·0, 5·4)**	**14·6 (13·2, 19·8)**

MAD: medically-attended diarrhea, mBGS: modified buffered glycerol saline.

* Incidence is calculated at the facility-level and summed to get country-level incidence and is the number of confirmed *Shigella* watery diarrhea cases per 100 child-years plus the number of confirmed *Shigella* dysentery cases per 100 child-years at risk. Observed incidence is not adjusted, estimated incidence at EFGH facilities adjusts incidence by accounting for children who were not enrolled and therefore not tested for *Shigella* but were eligible, estimating cases in the catchment area seeking care additionally adjusts incidence for the percentage who sought care at EFGH facilities among all care seeking, and the adjusted incidence is additionally adjusted for children in the catchment area who reported diarrhea of similar severity to facility-enrolled cases but did not report seeking care. 95% confidence intervals were generated using bootstrapping. Details of incidence rate calculations are shown in Table S6.

† Includes isolates from rectal swabs transported in mBGS or Cary-Blair media.

‡ Totals are the average of country-level estimates.

§ Defined as an ipaH cycle threshold (Ct) below the attributable cutoff.

Table S10. *Shigella* diarrhea severity-stratified adjusted incidence of MAD by culture and qPCR, by EFGH country.

	Adjusted *Shigella* incidence per 100 child-years (95% CI)*															
	**Bangladesh**		**Kenya**		**Malawi**		**Mali**		**Pakistan**		**Peru**		**The Gambia**		**Total**	
**Strata**	**Culture**	**qPCR†**	**Culture**	**qPCR†**	**Culture**	**qPCR†**	**Culture**	**qPCR†**	**Culture**	**qPCR†**	**Culture**	**qPCR†**	**Culture**	**qPCR†**	**Culture**	**qPCR†**
**Age and enrollment (months)**																
6-8	3·3 (1·7, 5·9)	8·7 (5·8, 14·3)	2·1 (0·3, 5·4)	4·8 (1·7, 10·5)	1·2 (0·2, 2·7)	0·4 (0·0, 1·5)	1·1 (0·0, 3·5)	9·8 (4·3, 21·2)	2·1 (1·1, 3·8)	7·2 (4·4, 12·0)	19·1 (7·9, 44·7)	32·2 (16·3, 69·0)	3·7 (0·9, 7·8)	10·9 (5·2, 19·9)	**4·7 (2·9, 8·5)**	**10·6 (7·9, 16·8)**
9-11	5·0 (2·6, 8·8)	12·8 (8·9, 20·3)	3·0 (0·5, 8·0)	7·9 (3·6, 17·0)	1·4 (0·4, 2·9)	3·9 (1·9, 7·3)	2·8 (0·3, 7·7)	13·7 (6·6, 32·4)	6·6 (3·6, 11·1)	14·6 (9·6, 21·7)	19·0 (8·5, 47·1)	22·4 (11·1, 52·8)	4·4 (1·5, 10·3)	20·9 (11·9, 37·5)	**6·0 (4·4, 10·5)**	**13·7 (11·2, 20·0)**
12-17	6·5 (4·5, 10·4)	13·0 (9·5, 19·3)	5·7 (3·0, 10·9)	13·9 (8·7, 23·6)	2·2 (1·1, 3·7)	2·5 (1·4, 4·4)	5·2 (2·8, 10·6)	19·6 (11·8, 38·0)	6·6 (4·3, 9·7)	14·4 (10·3, 20·2)	10·7 (6·2, 23·7)	25·2 (15·5, 51·6)	10·0 (5·5, 17·1)	30·1 (20·2, 48·6)	**6·7 (5·7, 9·5)**	**17·0 (14·7, 22·9)**
18-23	8·1 (5·3, 13·3)	15·8 (11·1, 23·9)	6·8 (3·6, 13·3)	14·8 (8·9, 25·9)	4·1 (2·4, 7·0)	6·6 (4·3, 10·6)	11·8 (7·2, 24·4)	31·3 (19·8, 61·2)	6·4 (3·9, 9·8)	15·9 (11·5, 22·5)	17·2 (8·9, 39·5)	36·0 (21·5, 80·4)	17·9 (10·9, 31·0)	53·5 (36·2, 84·1)	**10·3 (8·6, 14·9)**	**24·8 (21·4, 34·9)**
24-35	5·5 (3·3, 9·8)	9·2 (6·0, 15·8)	6·2 (3·5, 11·9)	10·4 (6·6, 19·2)	3·0 (1·8, 5·2)	3·2 (2·0, 5·6)	3·0 (1·4, 6·6)	8·6 (5·2, 17·0)	2·6 (1·5, 4·1)	6·0 (4·1, 8·6)	5·8 (2·9, 16·4)	14·0 (7·9, 35·6)	8·2 (5·2, 13·6)	18·9 (12·7, 29·1)	**4·9 (4·1, 7·0)**	**10·0 (8·7, 14·4)**
**Diarrhea severity definitions**																
**Dysentery**	1·7 (1·2, 2·8)	2·5 (1·8, 4·2)	1·5 (0·8, 2·9)	2·5 (1·5, 4·5)	0·4 (0·2, 0·8)	0·6 (0·3, 1·0)	0·7 (0·3, 1·7)	1·3 (0·7, 2·7)	1·1 (0·8, 1·6)	2·0 (1·5, 2·8)	2·8 (1·7, 6·6)	4·6 (3·1, 10·3)	3·6 (2·4, 6·0)	6·6 (4·5, 10·7)	**1·7 (1·4, 2·5)**	**2·9 (2·4, 4·2)**
**Watery diarrhea**	4·2 (3·1, 6·6)	9·1 (6·9, 13·4)	4·0 (2·5, 6·8)	8·7 (5·8, 14·4)	2·2 (1·5, 3·7)	2·9 (2·1, 4·6)	4·2 (2·7, 8·2)	14·5 (9·6, 28·4)	3·3 (2·5, 4·6)	8·4 (6·6, 11·2)	8·9 (6·0, 18·0)	18·6 (13·2, 38·2)	5·9 (3·8, 9·3)	20·2 (14·2, 30·8)	**4·7 (4·1, 6·4)**	**11·8 (10·5, 15·8)**
**Hospitalized**	0·3 (0·2, 0·7)	0·6 (0·4, 1·1)	0·1 (0·0, 0·4)	0·2 (<0·1, 0·7)	<0·1 (0·0, 0·1)	0·0 (0·0, 0·0)	0·0 (0·0, 0·0)	0·1 (0·0, 0·4)	<0·1 (0·0, 0·1)	0·1 (0·0, 0·2)	0·0 (0·0, 0·0)	0·2 (0·0, 0·8)	0·2 (<0·1, 0·6)	0·8 (0·4, 1·5)	**0·1 (0·1, 0·2)**	**0·3 (0·2, 0·5)**
**GEMS‡**																
LSD	3·8 (2·8, 6·1)	8·2 (6·3, 12·2)	1·6 (0·8, 3·1)	4·0 (2·5, 6·7)	2·2 (1·5, 3·6)	2·8 (2·0, 4·4)	3·7 (2·4, 7·4)	13·2 (8·7, 25·9)	2·9 (2·1, 4·0)	7·2 (5·6, 9·7)	1·0 (0·4, 2·5)	4·5 (2·3, 10·2)	5·6 (3·6, 8·8)	18·9 (13·1, 28·5)	**3·0 (2·5, 3·9)**	**8·4 (7·3, 11·1)**
MSD	2·1 (1·5, 3·3)	3·4 (2·6, 5·3)	3·9 (2·4, 6·7)	7·2 (4·8, 12·1)	0·5 (0·3, 0·9)	0·7 (0·4, 1·2)	1·2 (0·6, 2·6)	2·5 (1·6, 5·1)	1·6 (1·1, 2·2)	3·2 (2·4, 4·3)	10·7 (7·7, 22·8)	18·7 (13·5, 37·3)	3·9 (2·6, 6·5)	8·0 (5·6, 12·7)	**3·4 (2·9, 5·3)**	**6·2 (5·4, 9·5)**
***Shigella* mortality score§**																
Mild (<6 points)	5·4 (4·0, 8·4)	10·9 (8·5, 16·4)	4·8 (3·0, 8·3)	9·8 (6·7, 16·2)	2·6 (1·8, 4·3)	3·5 (2·5, 5·4)	4·7 (3·1, 9·3)	15·2 (10·1, 29·8)	3·8 (2·9, 5·3)	9·3 (7·3, 12·3)	5·3 (3·4, 11·1)	12·9 (8·8, 27·3)	9·2 (6·2, 14·3)	26·1 (18·5, 39·7)	**5·1 (4·6, 6·8)**	**12·5 (11·2, 16·6)**
Moderate (6-8 points)	0·4 (0·2, 0·8)	0·5 (0·3, 1·0)	0·6 (0·2, 1·2)	1·1 (0·6, 2·0)	<0·1 (0·0, <0·1)	<0·1 (0·0, <0·1)	0·2 (<0·1, 0·5)	0·5 (0·2, 1·1)	0·5 (0·3, 0·9)	1·0 (0·7, 1·5)	6·2 (4·3, 13·2)	10·1 (7·0, 21·5)	0·1 (0·0, 0·4)	0·3 (0·1, 0·7)	**1·2 (0·9, 2·2)**	**1·9 (1·5, 3·6)**
Severe (9+ points)	0·1 (<0·1, 0·3)	0·1 (<0·1, 0·3)	0·1 (0·0, 0·4)	0·3 (0·1, 0·8)	<0·1 (0·0, 0·1)	0·0 (0·0, 0·0)	0·0 (0·0, 0·0)	0·1 (0·0, 0·4)	<0·1 (0·0, 0·1)	0·1 (0·0, 0·2)	0·2 (0·0, 0·8)	0·2 (0·0, 0·8)	0·2 (<0·1, 0·4)	0·4 (0·1, 1·0)	**0·1 (<0·1, 0·2)**	**0·2 (0·1, 0·3)**
**Modified Vesikari score (MVS)‖**																
Mild (0-8 points)	4·2 (3·0, 6·6)	8·5 (6·4, 13·0)	3·1 (1·8, 5·3)	5·7 (3·6, 9·5)	2·2 (1·4, 3·6)	2·7 (1·9, 4·3)	3·9 (2·5, 8·0)	13·6 (9·0, 27·2)	3·0 (2·2, 4·2)	7·1 (5·5, 9·3)	5·6 (3·2, 12·2)	12·1 (7·7, 26·8)	8·0 (5·4, 12·5)	21·5 (15·0, 32·5)	**4·3 (3·7, 5·9)**	**10·2 (9·0, 13·8)**
Moderate (9-10 points)	0·4 (0·2, 0·8)	1·0 (0·7, 1·7)	1·4 (0·8, 2·6)	2·8 (1·7, 5·0)	0·3 (0·2, 0·6)	0·5 (0·3, 0·9)	0·5 (0·2, 1·1)	1·1 (0·6, 2·3)	0·8 (0·5, 1·2)	1·9 (1·3, 2·8)	3·5 (2·2, 7·6)	5·3 (3·5, 11·3)	0·5 (0·2, 1·0)	2·4 (1·5, 3·9)	**1·1 (0·9, 1·7)**	**2·2 (1·8, 3·3)**
Severe (11+ points)	1·2 (0·9, 2·1)	2·0 (1·5, 3·4)	1·0 (0·5, 2·0)	2·7 (1·7, 4·8)	0·2 (0·1, 0·3)	0·3 (0·1, 0·5)	0·5 (0·2, 1·1)	1·0 (0·6, 2·2)	0·6 (0·4, 1·0)	1·4 (0·9, 1·9)	2·6 (1·7, 5·9)	5·8 (4·1, 12·9)	1·0 (0·6, 1·8)	3·0 (1·9, 4·8)	**1·0 (0·9, 1·6)**	**2·3 (2·0, 3·5)**
**Moderate or severe diarrhea or dysentery¶**	2·7 (2·0, 4·2)	4·8 (3·8, 7·3)	3·4 (2·1, 5·9)	6·9 (4·6, 11·4)	0·8 (0·5, 1·3)	1·1 (0·8, 1·9)	1·5 (0·9, 3·0)	3·1 (2·0, 6·1)	1·9 (1·4, 2·7)	4·3 (3·4, 5·8)	7·0 (5·1, 14·5)	13·0 (9·5, 26·9)	4·3 (2·9, 6·9)	10·6 (7·5, 16·3)	**3·1 (2·7, 4·5)**	**6·3 (5·6, 9·0)**
**Clark score****																
Mild (2-8 points)	4·1 (2·9, 6·3)	8·6 (6·6, 13·2)	4·3 (2·6, 7·4)	8·6 (5·7, 14·3)	2·4 (1·7, 4·0)	3·0 (2·1, 4·6)	4·5 (3·0, 9·0)	15·0 (10·0, 29·3)	3·4 (2·5, 4·7)	8·0 (6·3, 10·8)	9·8 (6·5, 20·5)	18·7 (13·2, 38·5)	8·0 (5·4, 12·4)	22·7 (16·1, 34·0)	**5·2 (4·5, 7·2)**	**12·1 (10·8, 16·5)**
Severe (9+ points)	1·8 (1·3, 3·0)	3·0 (2·3, 4·6)	1·2 (0·7, 2·2)	2·6 (1·6, 4·5)	0·2 (0·1, 0·5)	0·5 (0·3, 0·9)	0·4 (0·1, 0·8)	0·7 (0·4, 1·5)	1·0 (0·7, 1·5)	2·4 (1·7, 3·2)	1·9 (1·3, 4·8)	4·5 (3·1, 10·4)	1·5 (0·9, 2·7)	4·2 (2·7, 6·9)	**1·2 (1·0, 1·7)**	**2·6 (2·3, 3·7)**
**MAL-ED score††**																
Non-severe (0-5 points)	3·2 (2·3, 5·1)	6·5 (4·9, 9·9)	2·2 (1·2, 4·0)	4·0 (2·5, 6·8)	2·0 (1·4, 3·5)	2·4 (1·7, 3·8)	3·4 (2·2, 7·1)	12·4 (8·0, 24·7)	2·2 (1·5, 3·2)	5·2 (3·9, 7·0)	3·3 (1·6, 7·8)	8·6 (5·1, 18·8)	5·2 (3·3, 8·2)	16·6 (11·5, 25·0)	**3·1 (2·7, 4·2)**	**8·0 (6·9, 10·8)**
Severe (6+ points)	2·7 (2·0, 4·3)	5·1 (3·9, 7·5)	3·3 (2·1, 5·8)	7·2 (4·8, 12·2)	0·6 (0·4, 1·0)	1·1 (0·7, 1·7)	1·4 (0·8, 2·9)	3·4 (2·2, 6·4)	2·2 (1·7, 3·1)	5·2 (4·1, 7·0)	8·4 (5·9, 17·5)	14·7 (10·7, 29·6)	4·3 (2·8, 6·9)	10·3 (7·2, 16·1)	**3·3 (2·8, 4·9)**	**6·7 (6·0, 9·7)**
**Vaccine targets**																
**Among all MAD**																
Bivalent (*S. flexneri* 2a or *S. sonnei*)	3·3 (2·4, 5·1)	4·3 (3·2, 6·5)	1·9 (1·1, 3·5)	2·7 (1·6, 4·6)	1·3 (0·8, 2·1)	1·1 (0·7, 1·8)	1·6 (0·9, 3·5)	3·4 (2·0, 6·8)	2·0 (1·4, 2·9)	3·5 (2·6, 4·8)	7·7 (5·3, 16·2)	13·5 (9·3, 27·3)	3·8 (2·5, 6·3)	8·0 (5·4, 12·4)	**3·1 (2·7, 4·6)**	**5·2 (4·6, 7·6)**
Quadrivalent (*S. flexneri* 2a, 3a, or 6 or *S. sonnei*)	4·1 (3·1, 6·3)	4·8 (3·7, 7·4)	4·0 (2·5, 7·0)	4·9 (3·2, 8·3)	1·6 (1·1, 2·6)	1·5 (1·0, 2·5)	2·9 (1·8, 5·9)	5·5 (3·5, 11·0)	2·4 (1·7, 3·4)	4·5 (3·5, 6·2)	8·0 (5·4, 17·3)	13·7 (9·5, 28·1)	6·1 (4·0, 9·6)	12·1 (8·4, 18·8)	**4·2 (3·7, 5·8)**	**6·7 (5·9, 9·5)**
Quadrivalent (*S. flexneri* 1b, 2a, or 3a or *S. sonnei*)	3·9 (2·9, 6·1)	4·5 (3·4, 6·8)	2·6 (1·5, 4·6)	3·7 (2·3, 6·3)	1·3 (0·9, 2·2)	1·2 (0·8, 2·0)	2·1 (1·3, 4·3)	4·6 (2·9, 9·1)	2·3 (1·7, 3·3)	4·9 (3·8, 6·7)	8·2 (5·6, 17·8)	15·0 (10·6, 30·5)	5·8 (3·8, 9·2)	10·1 (7·0, 15·6)	**3·7 (3·3, 5·3)**	**6·3 (5·6, 9·0)**
**Among GEMS MSD**																
Bivalent (*S. flexneri* 2a or *S. sonnei*)	1·1 (0·7, 1·8)	1·5 (1·0, 2·4)	1·2 (0·6, 2·3)	1·5 (0·8, 2·7)	0·3 (0·1, 0·6)	0·2 (0·1, 0·5)	0·4 (0·2, 1·0)	0·8 (0·4, 1·7)	0·7 (0·4, 1·0)	1·0 (0·7, 1·5)	6·9 (4·6, 14·8)	10·5 (7·4, 21·6)	1·5 (0·9, 2·6)	2·8 (1·8, 4·5)	**1·7 (1·4, 2·9)**	**2·6 (2·1, 4·3)**
Quadrivalent (*S. flexneri* 2a, 3a, or 6 or *S. sonnei*)	1·5 (1·0, 2·5)	1·6 (1·1, 2·6)	2·8 (1·7, 4·9)	3·0 (1·9, 5·4)	0·4 (0·2, 0·6)	0·3 (0·1, 0·6)	0·8 (0·4, 1·8)	1·2 (0·6, 2·7)	0·8 (0·5, 1·2)	1·4 (1·0, 2·1)	7·2 (4·9, 15·6)	10·7 (7·6, 22·0)	2·3 (1·4, 3·8)	3·9 (2·7, 6·3)	**2·2 (1·9, 3·6)**	**3·2 (2·7, 5·1)**
Quadrivalent (*S. flexneri* 1b, 2a, or 3a or *S. sonnei*)	1·4 (0·9, 2·3)	1·5 (1·1, 2·5)	1·5 (0·8, 2·8)	2·2 (1·3, 3·9)	0·3 (0·1, 0·6)	0·3 (0·1, 0·5)	0·7 (0·3, 1·5)	1·1 (0·6, 2·4)	0·8 (0·6, 1·2)	1·4 (1·0, 2·0)	7·4 (5·1, 15·8)	11·7 (8·3, 24·1)	2·4 (1·5, 4·0)	3·5 (2·4, 5·6)	**2·1 (1·7, 3·4)**	**3·1 (2·6, 5·1)**
**Among moderate or severe diarrhea by the modified Vesikari score and/or dysentery**																
Bivalent (*S. flexneri* 2a or *S. sonnei*)	1·3 (1·0, 2·1)	1·9 (1·3, 3·0)	1·0 (0·5, 1·9)	1·4 (0·8, 2·5)	0·4 (0·2, 0·7)	0·3 (0·2, 0·7)	0·5 (0·2, 1·1)	0·9 (0·5, 2·0)	0·8 (0·5, 1·3)	1·5 (1·0, 2·1)	4·1 (2·7, 8·8)	7·0 (4·8, 15·6)	1·5 (0·8, 2·5)	3·2 (2·1, 5·1)	**1·4 (1·2, 2·2)**	**2·3 (2·0, 3·7)**
Quadrivalent (*S. flexneri* 2a, 3a, or 6 or *S. sonnei*)	1·9 (1·4, 3·0)	2·2 (1·6, 3·5)	2·4 (1·5, 4·3)	2·9 (1·9, 5·2)	0·5 (0·3, 0·9)	0·4 (0·2, 0·8)	0·8 (0·4, 1·7)	1·5 (0·9, 3·1)	1·0 (0·7, 1·5)	1·9 (1·4, 2·7)	4·3 (3·0, 9·4)	7·2 (4·9, 16·1)	2·4 (1·4, 4·0)	4·9 (3·3, 7·8)	**1·9 (1·6, 2·9)**	**3·0 (2·6, 4·6)**
Quadrivalent (*S. flexneri* 1b, 2a, or 3a or *S. sonnei*)	1·7 (1·3, 2·8)	2·0 (1·4, 3·2)	1·4 (0·8, 2·6)	2·1 (1·3, 3·8)	0·4 (0·2, 0·8)	0·4 (0·2, 0·8)	0·8 (0·4, 1·8)	1·4 (0·8, 3·1)	1·0 (0·6, 1·5)	1·9 (1·4, 2·8)	4·6 (3·2, 9·8)	7·9 (5·5, 17·8)	2·5 (1·5, 4·2)	4·3 (2·8, 6·9)	**1·8 (1·5, 2·7)**	**2·9 (2·4, 4·4)**

GEMS: Global Enteric Multicenter Study, LSD: less-severe diarrhea, MAD: medically-attended diarrhea, MAL-ED: Malnutrition and Enteric Disease Study, MSD: moderate-to-severe diarrhea, MVS: modified Vesikari score.

* Incidence is calculated at the facility-level and summed to get country-level incidence and is the number of confirmed *Shigella* watery diarrhea cases per 100 child-years plus the number of confirmed *Shigella* dysentery cases per 100 child-years at risk. Incidence is adjusted by the percentage of children potentially eligible but not enrolled (stratified by facility and type of diarrhea) as well as for healthcare seeking from the Healthcare Utilization Survey. 95% confidence intervals were generated using bootstrapping.

† Defined as an ipaH cycle threshold below the attributable cutoff.

‡ Defined as in Kotloff, Lancet GH, 2019. Moderate-to-severe diarrhea (MSD) defined as presenting to a health facility with diarrhea and severe or some dehydration (by WHO criteria), visible blood in stool, or inpatient admission. Less-severe-diarrhea (LSD) defined as presenting to a health facility without MSD.

§ Defined as in Pavlinac, CID, 2021. Duration of diarrhea through day of presentation: 1-3 days (0 point), 4-5 days (2 points), ≥6 days (3 points); WHO-defined dehydration categories: severe (8 points), some (4 points), none (0 points); inpatient admission (5 points).

‖ Defined as in PATH Vesikari Clinical Severity Scoring System Manual: Duration of diarrhea: 1-4 days (1 point), 5 days (2 points) ≥6 days (3 points); Max # of stool in 24 hour period: 1-3 (1 point), 4-5 (2 points), ≥6 (3 points); Duration of vomiting: 1 day (1 point), 2 days (2 points), ≥3 days (3 points); max # of vomiting episodes in 24 hour period: 1 (1 point), 2-4 (2 points), ≥5 (3 points); Axillary temperature 36·6-37·9°C (1 point), 38·0-38·4°C (2 points), ≥38·5°C (3 points); dehydration 1-5% (2 points), ≥6% (3 points); treatment: rehydration (1 point), hospitalization (2 points).

¶ Defined as a MVS of 9+ or presence of visible blood in stool.

** Defined as in Clark, JID, 1988. Duration of diarrhea: 1-4 days (1 point), 5-7 days (2 points), >7 days (3 points); Max # of stool in 24 hour period: 2-4 (1 point), 5-7 (2 points), >7 (3 points); Duration of vomiting: 2 days (1 point), 3-5 days (2 points), >5 days (3 points); max # of vomiting episodes in 24 hour period: 1-3 (1 point), 4-6 (2 points), >6 (3 points); Duration of reported fever: 1-2 days (1 point), 3-4 days (2 points), ≥5 days (3 points); Rectal temperature 38·0-38·2°C (1 point), 38·3-38·7°C (2 points), ≥38·8°C (3 points); behavioral signs: Irritable/less playful (1 point), Lethargic/listless (2 points), Seizures (3 points).

†† Defined as in Lee, J Pediatr Gastroenterol Nutr., 2016: Duration of diarrhea: 2-4 days (1 point), 5-7 days (2 points), ≥8 days (3 points); Max # of stool in 24 hour period: <5 loose stools/24 hours (1 point), 5-7 loose stools/24 hours (2 points), ≥8 stools/24 hours (3 points); Duration of vomiting: 1 day (1 point), 2 days (2 points), ≥3 days (3 points); duration of reported fever: 1+ days (1 point); Confirmed temperature: ≥37·5°C (confirmed by field worker) (2 points); Dehydration: Some (2 points), severe (3 points).

Figure S2. Seasonality of adjusted *Shigella* MAD incidence by EFGH country site and laboratory method.

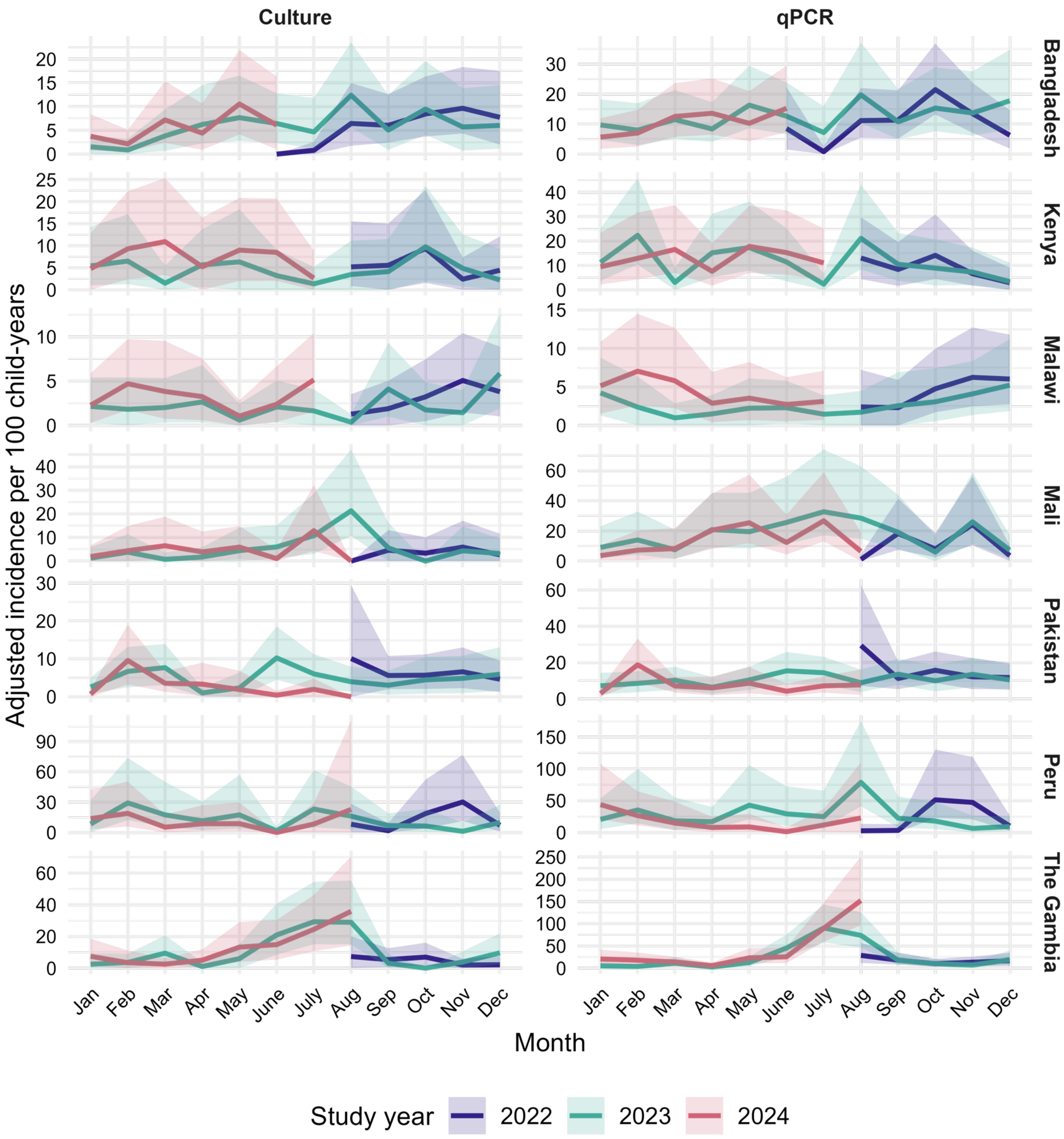

Adjusted incidence was defined as the estimated number of *Shigella* MAD cases per 100 child-years accounting for children who were not enrolled and therefore not tested for *Shigella* but were eligible as well as for children in the catchment area who reported diarrhea of similar severity to facility-enrolled cases but did not report seeking care at an EFGH facility. Adjusted incidence was computed for each month of study enrollment of interest by dividing the number of enrolled participants who tested positive for *Shigella* for culture or who were below the attributable Ct threshold by qPCR by the estimated child-years at risk residing in catchment area. 95% confidence intervals were generated using bootstrapping.

Table S11. Site-specific *Shigella* species and serotype distribution by culture and qPCR.

	Bangladesh		Kenya		Malawi		Mali		Pakistan		Peru		The Gambia		Total	
	Culture*	qPCR†	Culture*	qPCR†	Culture*	qPCR†	Culture*	qPCR†	Culture*	qPCR†	Culture*	qPCR†	Culture*	qPCR†	Culture*	qPCR†
***Shigella* serogroup: n/N (%)**																
S. boydii	25/200 (12·5%)	-	6/83 (7·2%)	-	12/99 (12·1%)	-	13/82 (15·9%)	-	19/160 (11·9%)	-	0/114 (0·0%)	-	8/146 (5·5%)	-	**83/884 (9·4%)**	**-**
S. dysenteriae	10/200 (5·0%)	-	2/83 (2·4%)	-	9/99 (9·1%)	-	2/82 (2·4%)	-	10/160 (6·2%)	-	1/114 (0·9%)	-	1/146 (0·7%)	-	**35/884 (4·0%)**	**-**
S. flexneri	95/200 (47·5%)	121/379 (31·9%)	48/83 (57·8%)	72/173 (41·6%)	38/99 (38·4%)	43/134 (32·1%)	54/82 (65·9%)	96/238 (40·3%)	85/160 (53·1%)	133/361 (36·8%)	80/114 (70·2%)	111/228 (48·7%)	97/146 (66·4%)	180/404 (44·6%)	**497/884 (56·2%)**	**756/1917 (39·4%)**
S. sonnei	67/200 (33·5%)	86/379 (22·7%)	24/83 (28·9%)	30/173 (17·3%)	40/99 (40·4%)	30/134 (22·4%)	13/82 (15·9%)	28/238 (11·8%)	42/160 (26·2%)	69/361 (19·1%)	33/114 (28·9%)	66/228 (28·9%)	38/146 (26·0%)	77/404 (19·1%)	**257/884 (29·1%)**	**386/1917 (20·1%)**
Undetermined‡	3/200 (1·5%)	172/379 (45·4%)	3/83 (3·6%)	71/173 (41·0%)	0/99 (0·0%)	61/134 (45·5%)	0/82 (0·0%)	114/238 (47·9%)	4/160 (2·5%)	159/361 (44·0%)	0/114 (0·0%)	51/228 (22·4%)	2/146 (1·4%)	147/404 (36·4%)	**12/884 (1·4%)**	**775/1917 (40·4%)**
***S. flexneri* serotype/subserotype: n/N (%)**																
1a	4/95 (4·2%)	3/121 (2·5%)	1/48 (2·1%)	0/72 (0·0%)	0/38 (0·0%)	0/43 (0·0%)	0/54 (0·0%)	0/96 (0·0%)	2/85 (2·4%)	3/133 (2·3%)	9/80 (11·2%)	14/111 (12·6%)	0/97 (0·0%)	0/180 (0·0%)	**16/497 (3·2%)**	**20/756 (2·6%)**
1b	1/95 (1·1%)	0/121 (0·0%)	3/48 (6·2%)	7/72 (9·7%)	1/38 (2·6%)	3/43 (7·0%)	7/54 (13·0%)	14/96 (14·6%)	9/85 (10·6%)	22/133 (16·5%)	3/80 (3·8%)	13/111 (11·7%)	24/97 (24·7%)	17/180 (9·4%)	**48/497 (9·7%)**	**76/756 (10·1%)**
1d	0/95 (0·0%)	0/121 (0·0%)	0/48 (0·0%)	0/72 (0·0%)	0/38 (0·0%)	0/43 (0·0%)	0/54 (0·0%)	1/96 (1·0%)	0/85 (0·0%)	1/133 (0·8%)	0/80 (0·0%)	0/111 (0·0%)	0/97 (0·0%)	0/180 (0·0%)	**0/497 (0·0%)**	**2/756 (0·3%)**
2a	50/95 (52·6%)	63/121 (52·1%)	6/48 (12·5%)	13/72 (18·1%)	5/38 (13·2%)	8/43 (18·6%)	15/54 (27·8%)	26/96 (27·1%)	27/85 (31·8%)	47/133 (35·3%)	39/80 (48·8%)	60/111 (54·1%)	19/97 (19·6%)	46/180 (25·6%)	**161/497 (32·4%)**	**263/756 (34·8%)**
2b	0/95 (0·0%)	1/121 (0·8%)	2/48 (4·2%)	6/72 (8·3%)	0/38 (0·0%)	0/43 (0·0%)	4/54 (7·4%)	6/96 (6·2%)	0/85 (0·0%)	5/133 (3·8%)	11/80 (13·8%)	11/111 (9·9%)	1/97 (1·0%)	5/180 (2·8%)	**18/497 (3·6%)**	**34/756 (4·5%)**
3a	22/95 (23·2%)	9/121 (7·4%)	7/48 (14·6%)	9/72 (12·5%)	1/38 (2·6%)	3/43 (7·0%)	2/54 (3·7%)	4/96 (4·2%)	8/85 (9·4%)	28/133 (21·1%)	5/80 (6·2%)	3/111 (2·7%)	5/97 (5·2%)	15/180 (8·3%)	**50/497 (10·1%)**	**71/756 (9·4%)**
3b	0/95 (0·0%)	2/121 (1·7%)	0/48 (0·0%)	0/72 (0·0%)	0/38 (0·0%)	0/43 (0·0%)	1/54 (1·9%)	0/96 (0·0%)	0/85 (0·0%)	3/133 (2·3%)	0/80 (0·0%)	3/111 (2·7%)	1/97 (1·0%)	3/180 (1·7%)	**2/497 (0·4%)**	**11/756 (1·5%)**
4a	8/95 (8·4%)	10/121 (8·3%)	7/48 (14·6%)	4/72 (5·6%)	15/38 (39·5%)	0/43 (0·0%)	7/54 (13·0%)	1/96 (1·0%)	6/85 (7·1%)	10/133 (7·5%)	11/80 (13·8%)	3/111 (2·7%)	4/97 (4·1%)	8/180 (4·4%)	**58/497 (11·7%)**	**36/756 (4·8%)**
4b	0/95 (0·0%)	0/121 (0·0%)	0/48 (0·0%)	0/72 (0·0%)	0/38 (0·0%)	0/43 (0·0%)	0/54 (0·0%)	0/96 (0·0%)	0/85 (0·0%)	0/133 (0·0%)	0/80 (0·0%)	4/111 (3·6%)	1/97 (1·0%)	2/180 (1·1%)	**1/497 (0·2%)**	**6/756 (0·8%)**
5a	0/95 (0·0%)	0/121 (0·0%)	0/48 (0·0%)	1/72 (1·4%)	0/38 (0·0%)	0/43 (0·0%)	0/54 (0·0%)	1/96 (1·0%)	0/85 (0·0%)	0/133 (0·0%)	0/80 (0·0%)	0/111 (0·0%)	0/97 (0·0%)	1/180 (0·6%)	**0/497 (0·0%)**	**3/756 (0·4%)**
5b	0/95 (0·0%)	19/121 (15·7%)	0/48 (0·0%)	0/72 (0·0%)	0/38 (0·0%)	0/43 (0·0%)	0/54 (0·0%)	1/96 (1·0%)	0/85 (0·0%)	0/133 (0·0%)	0/80 (0·0%)	0/111 (0·0%)	0/97 (0·0%)	1/180 (0·6%)	**0/497 (0·0%)**	**21/756 (2·8%)**
6	8/95 (8·4%)	9/121 (7·4%)	22/48 (45·8%)	23/72 (31·9%)	13/38 (34·2%)	15/43 (34·9%)	17/54 (31·5%)	28/96 (29·2%)	8/85 (9·4%)	7/133 (5·3%)	0/80 (0·0%)	0/111 (0·0%)	26/97 (26·8%)	45/180 (25·0%)	**94/497 (18·9%)**	**127/756 (16·8%)**
7a	-	5/121 (4·1%)	-	3/72 (4·2%)	-	0/43 (0·0%)	-	1/96 (1·0%)	-	6/133 (4·5%)	-	0/111 (0·0%)	-	2/180 (1·1%)	**-**	**17/756 (2·2%)**
X	0/95 (0·0%)	0/121 (0·0%)	0/48 (0·0%)	6/72 (8·3%)	1/38 (2·6%)	14/43 (32·6%)	1/54 (1·9%)	13/96 (13·5%)	0/85 (0·0%)	1/133 (0·8%)	0/80 (0·0%)	0/111 (0·0%)	0/97 (0·0%)	35/180 (19·4%)	**2/497 (0·4%)**	**69/756 (9·1%)**
Y	0/95 (0·0%)	0/121 (0·0%)	0/48 (0·0%)	0/72 (0·0%)	0/38 (0·0%)	0/43 (0·0%)	0/54 (0·0%)	0/96 (0·0%)	1/85 (1·2%)	0/133 (0·0%)	2/80 (2·5%)	0/111 (0·0%)	0/97 (0·0%)	0/180 (0·0%)	**3/497 (0·6%)**	**0/756 (0·0%)**
Non-typeable	2/95 (2·1%)	0/121 (0·0%)	0/48 (0·0%)	0/72 (0·0%)	2/38 (5·3%)	0/43 (0·0%)	0/54 (0·0%)	0/96 (0·0%)	24/85 (28·2%)	0/133 (0·0%)	0/80 (0·0%)	0/111 (0·0%)	16/97 (16·5%)	0/180 (0·0%)	**44/497 (8·9%)**	**0/756 (0·0%)**
***S. dysenteriae* serotype: n/N (%)**																
Type 1	0/10 (0·0%)	-	0/2 (0·0%)	-	0/9 (0·0%)	-	0/2 (0·0%)	-	0/10 (0·0%)	-	0/1 (0·0%)	-	0/1 (0·0%)	-	**0/35 (0·0%)**	**-**
Non-Type 1	10/10 (100·0%)	-	2/2 (100·0%)	-	9/9 (100·0%)	-	2/2 (100·0%)	-	10/10 (100·0%)	-	1/1 (100·0%)	-	1/1 (100·0%)	-	**35/35 (100·0%)**	**-**
**Vaccine targets: n/N (%)**																
Bivalent formulation§	117/200 (58·5%)	149/379 (39·3%)	30/83 (36·1%)	43/173 (24·9%)	45/99 (45·5%)	38/134 (28·4%)	28/82 (34·1%)	54/238 (22·7%)	69/160 (43·1%)	116/361 (32·1%)	72/114 (63·2%)	126/228 (55·3%)	57/146 (39·0%)	123/404 (30·4%)	**418/884 (47·3%)**	**649/1917 (33·9%)**
Quadrivalent formulation‖	147/200 (73·5%)	167/379 (44·1%)	59/83 (71·1%)	75/173 (43·4%)	59/99 (59·6%)	56/134 (41·8%)	47/82 (57·3%)	86/238 (36·1%)	85/160 (53·1%)	151/361 (41·8%)	77/114 (67·5%)	129/228 (56·6%)	88/146 (60·3%)	182/404 (45·0%)	**562/884 (63·6%)**	**846/1917 (44·1%)**
Alternate quadrivalent formulation¶	140/200 (70·0%)	158/379 (41·7%)	40/83 (48·2%)	59/173 (34·1%)	47/99 (47·5%)	44/134 (32·8%)	37/82 (45·1%)	72/238 (30·3%)	86/160 (53·8%)	166/361 (46·0%)	80/114 (70·2%)	142/228 (62·3%)	86/146 (58·9%)	154/404 (38·1%)	**516/884 (58·4%)**	**795/1917 (41·5%)**
**Vaccine targets including cross-protection: n/N (%)**																
Bivalent formulation**	129/200 (64·5%)	165/379 (43·5%)	40/83 (48·2%)	54/173 (31·2%)	60/99 (60·6%)	38/134 (28·4%)	40/82 (48·8%)	62/238 (26·1%)	77/160 (48·1%)	137/361 (38·0%)	103/114 (90·4%)	157/228 (68·9%)	63/146 (43·2%)	140/404 (34·7%)	**512/884 (57·9%)**	**753/1917 (39·3%)**
Quadrivalent formulation††	159/200 (79·5%)	202/379 (53·3%)	69/83 (83·1%)	92/173 (53·2%)	75/99 (75·8%)	70/134 (52·2%)	60/82 (73·2%)	108/238 (45·4%)	93/160 (58·1%)	173/361 (47·9%)	108/114 (94·7%)	164/228 (71·9%)	95/146 (65·1%)	238/404 (58·9%)	**659/884 (74·5%)**	**1047/1917 (54·6%)**
Alternate quadrivalent formulation‡‡	152/200 (76·0%)	193/379 (50·9%)	50/83 (60·2%)	76/173 (43·9%)	63/99 (63·6%)	58/134 (43·3%)	50/82 (61·0%)	94/238 (39·5%)	94/160 (58·8%)	188/361 (52·1%)	111/114 (97·4%)	177/228 (77·6%)	93/146 (63·7%)	210/404 (52·0%)	**613/884 (69·3%)**	**996/1917 (52·0%)**

* *S. flexneri* 7a is not assessed by culture.

† Defined as an ipaH cycle threshold (Ct) below the attributable cutoff. *S. boydii* and *S. dysentariae* are not assessed by qPCR.

‡ Undetermined by culture means any serotypes/subserotypes not listed in the table. Undetermined by qPCR means *Shigella* detected by qPCR but molecular criteria to assign species/serotype to *S. sonnei* or *S. flexneri* was not met or species not tested.

§ *S. flexneri* 2a or *S. sonnei*.

‖ *S. flexneri* 2a, 3a, or 6 or *S. sonnei*.

¶ *S. flexneri* 1b, 2a, or 3a or *S. sonnei*.

** *S. flexneri* 2a or *S. sonnei* plus cross-protection against *S. flexneri* 1a, 2b, 3b, 4a and 5a.

†† *S. flexneri* 2a, 3a or 6 or *S. sonnei* plus cross-protection against *S. flexneri* 1a, 2b, 3b, 4a, 4b, 5a, 5b and X.

‡‡ *S. flexneri* 1b, 2a or 3a or *S. sonnei* plus cross-protection against *S. flexneri* 1a, 2b, 3b, 4a, 4b, 5a, 5b and X.

Figure S3. Country-specific antimicrobial resistance to culture-confirmed *Shigella* isolates.

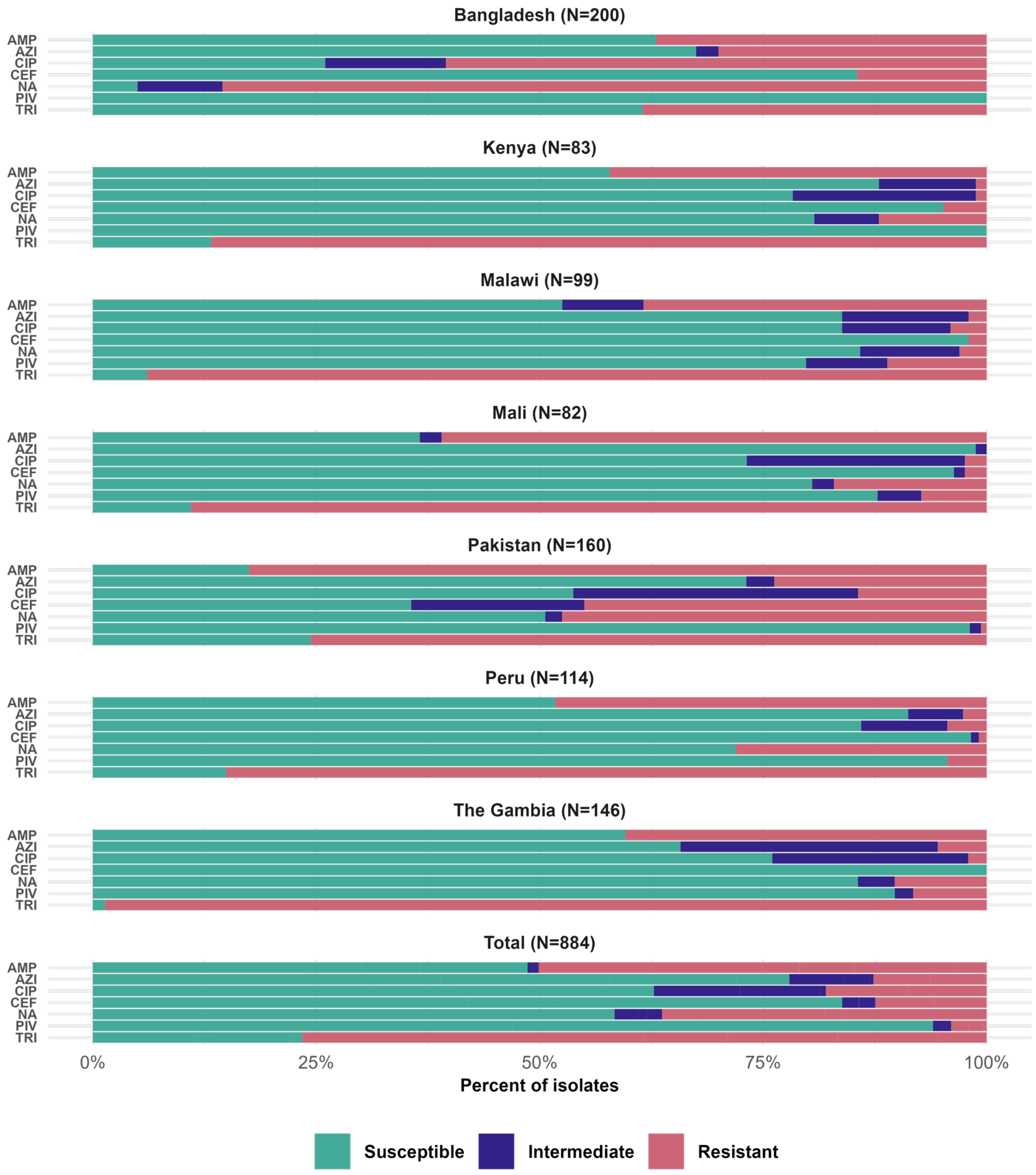

The percentage of *Shigella* culture-positive isolates resistant to antibiotics (AMP: ampicillin, AZI: azithromycin, CIP: ciprofloxacin, CTX: ceftriaxone, NA: nalidixic acid, PIV: pivmicellinam, and TRI-SMX: trimethroprim-sulfamethoxazole) was determined overall and for EFGH country site. Resistance was classified per CLSI guidelines as susceptible, intermediate or resistant.

Table S12. ICD-11 causes of death for all known mortality events occurring within the EFGH three-month follow-up period.

Country site	Time from enrollment to death (days)	Age at death (months)	*Shigella* at enrollment†	Cause of death*			
				Immediate (1a)	Distal (1b)	Most Distal (1c)	Underlying (2)
Bangladesh	20 days	6	*Shigella*	CA40·Z: Pneumonia, organism Unspecified	5B51: Wasting in infants, children, or adolescents		3A9Z: Anaemias or other erythrocyte disorders, unspecified, 1A02: Intestinal infections due to *Shigella*
Bangladesh	21 days	25	No *Shigella*	MH12: Other sudden death, cause unknown			
Kenya	19 days	8	*Shigella*	1A40·Z: Infectious gastroenteritis or colitis without specification of infectious agent			5B51: Wasting in infants, children, or adolescents, 1A02: Intestinal infections due to *Shigella*
Kenya	2 days	10	No *Shigella*	3A9Z: Anaemias or other erythrocyte disorders, unspecified	1F4Z: Malaria, unspecified		1F62: Ascariasis
Kenya	101 days	11	No *Shigella*	CA71·0: Pneumonitis due to inhalation of food or vomit	CA40·Z: Pneumonia, organism Unspecified	8D2Z: Cerebral palsy, unspecified	
Kenya	64 days	8	No *Shigella*	1F4Z: Malaria, unspecified			NB3Z: Injuries to the thorax, unspecified
Malawi	97 days	17	No *Shigella*	MG40·1: Hypovolaemic shock	5C70·0: Dehydration	1A00: Cholera	
Malawi	27 days	24	*Shigella*	MG40·1: Hypovolaemic shock	5C70·0: Dehydration	1A00: Cholera	
Malawi	68 days	31	No *Shigella*	CA40·Z: Pneumonia, organism unspecified	XN487: Human immunodeficiency virus	5B51: Wasting in infants, children, or adolescents	GB41: Nephrotic syndrome, 3A9Z: Anaemias or other erythrocyte disorders, unspecified
Mali	16 days	8	No *Shigella*	CA40·Z: Pneumonia, organism unspecified	1F03: Measles		
Mali	28 days	18	No *Shigella*	MH12: Other sudden death, cause unknown			
Mali	9 days	7	No *Shigella*	ME05.1: Diarrhoea			5B51: Wasting in infants, children, or adolescents
Mali	3 days	11	*Shigella*	1F4Z: Malaria, unspecified	3A9Z: Anaemias or other erythrocyte disorders, unspecified	5B51: Wasting in infants, children, or adolescents	
Pakistan	23 days	10	No *Shigella*	1G41: Sepsis with septic shock	CA40·Z: Pneumonia, organism unspecified	1F03: Measles	
Pakistan	32 days	22	No *Shigella*	CA71·0: Pneumonitis due to inhalation of food or vomit	8D2Z: Cerebral palsy, unspecified		
Pakistan	42 days	10	No *Shigella*	CA71·0: Pneumonitis due to inhalation of food or vomit			
The Gambia	22 days	8	*Shigella*	1G41: Sepsis with septic shock	EA88·0: Infectious dermatitis	5B51: Wasting in infants, children, or adolescents	
The Gambia	25 days	14	No *Shigella*	1G41: Sepsis with septic shock	CA40·Z: Pneumonia, organism Unspecified		
The Gambia	2 days	12	No *Shigella*	1G41: Sepsis with septic shock	5C70·0: Dehydration	1A40·Z: Infectious gastroenteritis or colitis without specification of infectious agent	3A9Z: Anaemias or other erythrocyte disorders, unspecified
The Gambia	13 days	16	No *Shigella*	1G41: Sepsis with septic shock	1D01·Z: Infectious meningitis, unspecified	CA40·Z: Pneumonia, organism unspecified	
The Gambia	1 days	12	No *Shigella*	CA40·Z: Pneumonia, organism unspecified	5C70·0: Dehydration	5B51: Wasting in infants, children, or adolescents	5A41: Hypoglycemia without association to diabetes
The Gambia	3 days	12	No *Shigella*	CA40·Z: Pneumonia, organism unspecified	1G41: Sepsis with septic shock	5B51: Wasting in infants, children, or adolescents	

ICD-11: International Classification of Diseases 11th Revision.

* International Classification of Diseases, Eleventh Revision (ICD-11), World Health Organization (WHO) 2019/2021 https://icd.who.int/browse11. Licensed under Creative Commons Attribution-NoDerivatives 3·0 IGO licence (CC BY-ND 3·0 IGO). Causes of death are agreed upon by a panel of ICD-11 trained clinicians using information from the child's death certificate (if available), caregiver interview (if available), and case notes from the child's care team. Missing cause of death are for deaths that have not yet been reviewed by the cause of death panel.

† By culture isolation or below the qPCR attributable cycle threshold cutoff.

Figure S4. Prevalence of qPCR-attributable enteric co-pathogens among children 6-35 months with qPCR-attributable or culture positive *Shigella* MAD, by site and diarrhea severity.

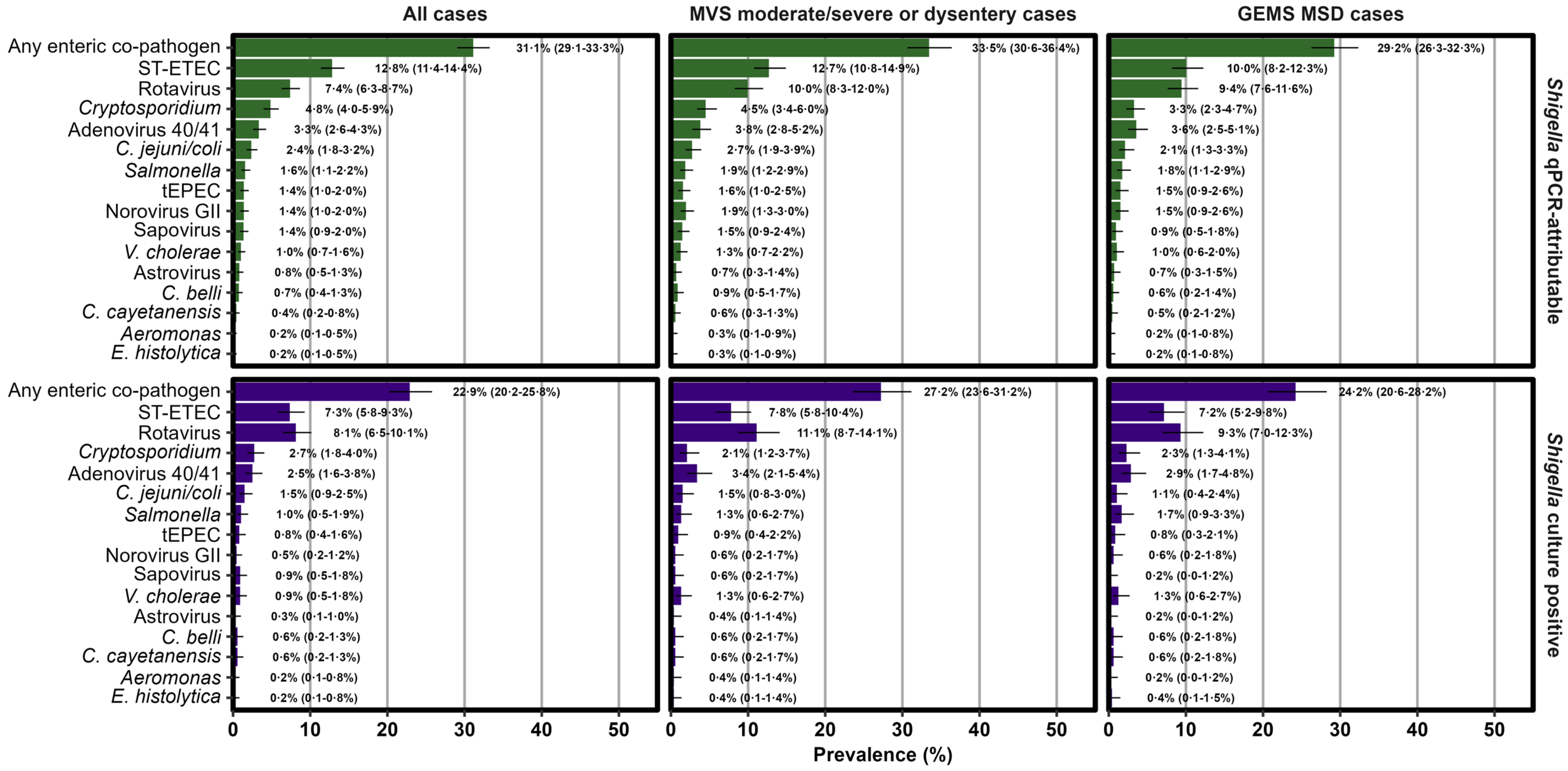

The percentage of samples who were attributable to enteric co-pathogens among *Shigella* qPCR-attributable or *Shigella* culture positive participant samples were calculated overall and by EFGH country site and stratified by diarrhea severity (all cases, moderate or severe diarrhea by the modified Vesikari score [MVS] or dysentery, and moderate-to-severe diarrhea [MSD] by GEMS). Enteric pathogens of interest included adenovirus 40/41, *Aeromonas*, astrovirus, *Cystoisospora belli* (*C. belli*), *Cyclospora cayatanensis* (*C. cayatanensis*), *Campylobacter jejuni/coli* (*C. jejuni/coli*), *Cryptosporidium*, *Entamoeba histolytica* (*E. histolytica*), norovirus GII, rotavirus, *Salmonella*, sapovirus, heat-stable enterotoxigenic *E. coli* (ST-ETEC), typical enteropathogenic *E. coli* (tEPEC) and *Vibrio cholerae* as well as a combined outcome of any attributable enteric pathogen. Sample sizes within each panel vary to due samples that did not yield a valid qPCR result for a particular target, however overall panel sample sizes are as follows: all cases (N=1870), MVS moderate or severe diarrhea or dysentery (N=1028) and GEMS MSD (N=859) among *Shigella* qPCR-attributable participants samples; and all cases (N=878), MVS moderate or severe diarrhea or dysentery (N=529) and GEMS MSD (N=479) among *Shigella* culture positive participants samples. Species are presented the order of prevalence among all *Shigella* qPCR-attributable cases and uncertainty is expressed as Wilson binomial 95% confidence intervals. The attributable cycle threshold (Ct) for each enteric co-pathogen of interest can be found in Table S4.

Table S13. *Shigella* incidence using a healthcare-seeking adjustment among diarrhea reported within the past seven days.

Country	*Shigella* incidence per 100 child-years (95% CI)* 14 days of recall (per SAP)†	7 days of recall (sensitivity analysis)†
**Confirmed by culture‡**		
Bangladesh	5·9	6·5
Kenya	5·5	6·0
Malawi	2·7	2·8
Mali	4·9	5·0
Pakistan	4·4	4·6
Peru	11·7	11·9
The Gambia	9·5	9·9
**Total§**	**6·4**	**6·7**
**Attributable by molecular diagnostics (qPCR)¶**		
Bangladesh	11·6	12·7
Kenya	11·2	12·0
Malawi	3·5	3·6
Mali	15·7	16·3
Pakistan	10·4	10·8
Peru	23·2	23·3
The Gambia	26·9	28·2
**Total§**	**14·7**	**15·3**

Ct: cycle threshold, MAD medically-attended diarrhea, mBGS: modified buffered glycerol saline, SAP: statistical analysis plan.

* Incidence is calculated at the facility-level and summed to get country-level incidence and is the number of confirmed *Shigella* watery diarrhea cases per 100 child-years plus the number of confirmed *Shigella* dysentery cases per 100 child-years at risk. Incidence is adjusted for children in the catchment area who reported diarrhea of similar severity to facility-enrolled cases but did not report seeking care. Details of incidence rate calculations are shown in Table S6.

† The healthcare seeking adjustment is determined using a propensity to seek care comparing enrolled children to children of a similar age and severity and the community to account for children who did not seek care. This adjustment is based on caregiver-reported diarrhea within the previous 14 days, whereas the sensitivity analysis applies the same propensity to seek care modelling approach but restricted to children who reported diarrhea in the previous seven days.

‡ Includes isolates from rectal swabs transported in mBGS or Cary-Blair media.

§ Totals are the average of country-level estimates.

¶ Defined as an ipaH cycle threshold (Ct) below the attributable cutoff.

Table S14. Characteristics of the population enumeration and healthcare utilization survey, by study site.

Indicator	Bangladesh	Kenya	Malawi	Mali	Pakistan	Peru	The Gambia	Total
**Population Enumeration**								
**Total clusters demarcated: n**	731	572	117	1046	6495	240	726	9927
**Study area: km^2**	5·0	573·4	6·3	7·4	46·4	14·2	695·2	1347·9
**Clusters enumerated: n (%)**								
Cluster enumerated	573 (78·4%)	152 (26·3%)	83 (70·9%)	319 (30·5%)	690 (10·6%)	180 (75·0%)	201 (27·7%)	2198 (22·1%)
No households present in the cluster	158 (21·6%)	5 (0·9%)	11 (9·4%)	29 (2·8%)	203 (3·1%)	56 (23·3%)	525 (72·3%)	987 (9·9%)
Could not enumerated cluster due to safety or other reasons	0 (0·0%)	0 (0·0%)	1 (0·9%)	10 (1·0%)	98 (1·5%)	3 (1·2%)	0 (0·0%)	112 (1·1%)
Not enumerated	0 (0·0%)	421 (72·8%)	22 (18·8%)	688 (65·8%)	5504 (84·7%)	1 (0·4%)	0 (0·0%)	6636 (66·8%)
**Total individuals enumerated: n**	396 004	70 372	72 729	67 711	214 513	83 108	145 781	1 050 218
**Children 6-35 months of age enumerated: n**	17 127	4601	4258	5921	10 024	3023	9655	54 609
**Caregiver reported children 6-35 months of age had diarrhea in the past two weeks: n (%)**								
Yes	816 (4·8%)	574 (12·5%)	299 (7·0%)	528 (8·9%)	1298 (12·9%)	240 (7·9%)	1098 (11·4%)	4853 (8·9%)
No	16 311 (95·2%)	4026 (87·5%)	3957 (92·9%)	5393 (91·1%)	8726 (87·1%)	2781 (92·0%)	8546 (88·5%)	49 740 (91·1%)
Don't know	0 (0·0%)	1 (<0·1%)	2 (<0·1%)	0 (0·0%)	0 (0·0%)	2 (0·1%)	11 (0·1%)	16 (<0·1%)
**Caregiver of child 6-35 months of age with diarrhea consented to HUS: n (%)**								
Yes, consented	816 (100·0%)	574 (100·0%)	280 (93·6%)	528 (100·0%)	1283 (98·8%)	235 (97·9%)	1078 (98·2%)	4794 (98·8%)
No, refused	0 (0·0%)	0 (0·0%)	10 (3·3%)	0 (0·0%)	11 (0·8%)	5 (2·1%)	16 (1·5%)	42 (0·9%)
No, caregiver not available for consent and no successful revisit	0 (0·0%)	0 (0·0%)	9 (3·0%)	0 (0·0%)	4 (0·3%)	0 (0·0%)	4 (0·4%)	17 (0·4%)
**Care-seeking for diarrhea: n (%)**								
Did not seek care	298 (36·5%)	155 (27·0%)	102 (36·4%)	38 (7·2%)	510 (39·8%)	128 (54·5%)	566 (52·5%)	1797 (37·5%)
Sought care at an EFGH facility, other outpatient or inpatient hospital or health center	176 (21·6%)	172 (30·0%)	138 (49·3%)	84 (15·9%)	707 (55·1%)	49 (20·9%)	405 (37·6%)	1731 (36·1%)
Sought care at other location*	342 (41·9%)	247 (43·0%)	40 (14·3%)	406 (76·9%)	66 (5·1%)	58 (24·7%)	107 (9·9%)	1266 (26·4%)
**Demographic indicators of children in the HUS**								
**Female sex: n (%)**	381 (46·7%)	244 (42·5%)	151 (53·9%)	237 (44·9%)	633 (49·3%)	105 (44·7%)	507 (47·0%)	2,258 (47·1%)
**Age (months): n (%)**								
6—8	118 (14·5%)	76 (13·2%)	24 (8·6%)	68 (12·9%)	145 (11·3%)	11 (4·7%)	104 (9·6%)	546 (11·4%)
9—11	131 (16·1%)	79 (13·8%)	49 (17·5%)	67 (12·7%)	156 (12·2%)	28 (11·9%)	135 (12·5%)	645 (13·5%)
12—17	204 (25·0%)	136 (23·7%)	78 (27·9%)	161 (30·5%)	331 (25·8%)	58 (24·7%)	252 (23·4%)	1220 (25·4%)
18—23	145 (17·8%)	123 (21·4%)	52 (18·6%)	112 (21·2%)	262 (20·4%)	60 (25·5%)	254 (23·6%)	1008 (21·0%)
24—35	218 (26·7%)	160 (27·9%)	77 (27·5%)	120 (22·7%)	389 (30·3%)	78 (33·2%)	333 (30·9%)	1375 (28·7%)
**Age in months: median (IQR)**	16 (10 - 24)	17 (11 - 24)	16 (11 - 24)	16 (11 - 23)	18 (12 - 25)	20 (14 - 26)	19 (12 - 25)	17 (12 - 24)
**Clinical characteristics**								
**Symptoms during diarrheal illness: n (%)**†								
Blood in stool	54 (6·6%)	47 (8·2%)	35 (12·5%)	15 (2·8%)	35 (2·7%)	24 (10·2%)	137 (12·7%)	347 (7·2%)
Irritable	328 (40·2%)	444 (77·4%)	191 (68·2%)	381 (72·2%)	950 (74·0%)	162 (68·9%)	579 (53·7%)	3035 (63·3%)
Very thirsty	629 (77·1%)	439 (76·5%)	172 (61·4%)	290 (54·9%)	1031 (80·4%)	206 (87·7%)	998 (92·6%)	3765 (78·5%)
Sunken eyes	238 (29·2%)	222 (38·7%)	77 (27·5%)	106 (20·1%)	228 (17·8%)	106 (45·1%)	365 (33·9%)	1342 (28·0%)
Wrinkled skin	101 (12·4%)	67 (11·7%)	45 (16·1%)	25 (4·7%)	122 (9·5%)	10 (4·3%)	97 (9·0%)	467 (9·7%)
Drinks eagerly, thirsty	652 (79·9%)	409 (71·3%)	212 (75·7%)	450 (85·2%)	1036 (80·7%)	195 (83·0%)	987 (91·6%)	3941 (82·2%)
Unable to drink or drank poorly	105 (12·9%)	109 (19·0%)	40 (14·3%)	10 (1·9%)	246 (19·2%)	14 (6·0%)	73 (6·8%)	597 (12·5%)
Lethargic, unconscious, or hard to stay awake	150 (18·4%)	34 (5·9%)	11 (3·9%)	2 (0·4%)	204 (15·9%)	16 (6·8%)	18 (1·7%)	435 (9·1%)

HUS: healthcare utilization survey, IQR: interquartile range.

* Health outpost, drug seller, pharmacist, traditional or religious healer, or other source.

† Column does not sum to total as surveyed participants may have reported multiple clinical symptoms.
