# Supplementary material for: The incidence and antimicrobial resistance of Shigella-attributable diarrhoea in young children in low-income and middle-income countries from the multicountry Enterics for Global Health (EFGH) Shigella Surveillance Study: a prospective, facility-based hybrid surveillance study
Source: Lancet Glob Health. 2026 Mar 11;14(5):e749–61. doi: 10.1016/S2214-109X(25)00534-0 (PMC13106044; doi:10.1016/S2214-109X(25)00534-0)
Supplement: Equitable Partnership Declaration [file mmc2.pdf]

THE LANCET
Global Health

Supplementary appendix 2

This Equitable Partnership Declaration (EPD) was submitted by the authors, and we reproduce it as supplied. It has not been peer reviewed. *The Lancet*'s editorial processes have not been applied to the EPD.

Supplement to: Yousafzai MT, Cornick J, Penataro Yori P, et al. The incidence and antimicrobial resistance of *Shigella*-attributable diarrhoea in young children in low-income and middle-income countries from the multicountry Enterics for Global Health (EFGH) *Shigella* Surveillance Study: a prospective, facility-based hybrid surveillance study. *Lancet Glob Health* 2026; published online March 11. https://doi.org/10.1016/S2214-109X(25)00534-0.

Equitable Partnership Declaration

If any questions do not apply to your study, please indicate "N/A" for "not applicable.
For more information on how to complete this form see the Information for Authors document.

Researcher considerations

1. Please detail the involvement that researchers who are based in the country or countries of study had during a) study design; b) clinical study processes, such as processing blood samples, prescribing medication, or patient recruitment; c) data interpretation; and d) manuscript preparation, commenting on all aspects. If they were not involved in any of these aspects, please explain why.

This should include a thorough description of their leadership roles in the study. Are local researchers named in the author list or the acknowledgements, or are they not mentioned at all (and, if not, why)? Please also describe the involvement of early career researchers based in the location of the study. Some of this information might be repeated from the Contributors section in the manuscript. Note: we adhere to ICMJE authorship criteria for naming authors on a paper.

**a) Study design:**<br><br>The EFGH study protocol was jointly developed across all seven consortium sites of Bangladesh, Kenya, Mali, Malawi, Pakistan, Peru, The Gambia during a planning phase funded by the Gates Foundation. The University of Washington coordination team organized bi-weekly protocol development working groups that were comprised of representatives from each of the country sites and coordinating bodies. The goals of these working groups were to 1) Co-develop the study protocol, standard operating procedures (SOPs), and case report forms (CRFs); 2) Promote and elevate scientific and operational ownership of the protocol across EFGH country sites; and 3) Build collaboration and knowledge sharing across the EFGH Consortium. Site-nominated working group members (which included junior investigators and staff from EFGH sites) participated in working groups focused on 4 study domains: Diarrhea case surveillance and follow up, population enumeration & healthcare utilization survey, laboratory methods and specimen collection, and data management. These working groups were intentionally sized to promote knowledge sharing and consensus building in an accessible environment. The working group model facilitated the development of study procedures and resources that leveraged learnings and expertise from site investigators and teams' robust research experiences. The primary outputs from these working groups included the final central protocol, CRFs, SOPs, and the central data management plan, along with other study resources such as study logs, training materials, and caregiver memory aids.
**b) Clinical study processes:**<br><br>Four trainees from 6 EFGH sites (Kenya, Malawi, Mali, The Gambia, Pakistan, Bangladesh) attended a Training of Trainers (ToT) in Nairobi, Kenya in March 2022. The training was facilitated by site trainers and members from the University of Washington and University of Virginia coordination teams. This was the first in-person meeting of EFGH study teams and the ToT model was chosen to build site leadership and capacity, and manage safe gathering during COVID. The goal of the 8-day training was to ensure comprehension of overall EFGH study protocol and procedures, create inter-site knowledge sharing and skill building opportunities, and equip ToT attendees with content and methods expertise to implement successful site-level down trainings following the ToT. Lead anthropometry trainers from the Kenya site successfully

executed the anthropometry standardization test to certify ToT attendees as gold-standard measurers. Training materials (facilitator guides, SOP/CRF manuals, slide decks, comprehension tests, evaluation and reporting templates) were provided to the ToTs for use at the ToT-led down trainings to ensure standardization of content and effective incorporation of adult learning principles to maximize accessibility and comprehension of training curriculum. Because of language barriers, a separate site-wide training was conducted at the Peru site in Iquitos by the UW coordination team using the same training content and resources.

In-person and virtual training sessions were conducted with all 7 EFGH site teams, during which approximately 30 microbiology lab personnel were trained by the University of Maryland, Baltimore (UMB Lab) team. Ahead of the trainings, the UMB team administered a site lab self-assessment aimed at understanding each site's lab resources, staffing, and familiarity with testing procedures. The microbiology trainings combined didactic and technical training (TSI Slant inoculation, MIO/MIU Media, LCD Testing, Serology Testing, Media Preparation), culminating with standardized proficiency testing among trained lab techs. Following each site visit, the UMB lab team produced a site visit report summarizing training activities and guidance for lab management improvement and technical feedback. The UMB team also oversaw central lab supply procurement and developed cross-Consortium lab materials including lab worksheets, logs, and quality assurance and control plans.

c) Data interpretation:

To facilitate data access and availability, the UW central data team, in collaboration with the Consortium data management working group made up of representatives from each country site, developed standing data reporting systems that produced site-specific monthly data reports, combined quarterly data reports, and the Data Read-Outs with preliminary results that were shared with the Gates Foundation.

As part of the EFGH Consortium's commitment to data accessibility and transparency, the central data team deployed the Consortium's "data dashboard." The dashboard displayed real-time study data including process and outcome indicators of interest (example dashboard visualizations below). EFGH consortium members were able to utilize figures such as the enrolment curve to monitor enrolment progress against a target, the anthropometry scatterplots to review erroneous values falling out of range, or the antibiotic resistance bar graphs to identify an early need for disseminating antibiotic resistance results to stakeholders. Regular review of the dashboard was integrated into daily site workflows and quality control measures and leveraged throughout the active study period to inform operational and scientific decision making to ensure a data-drive approach.

We also convened a statistical analysis plan working group that met monthly from Jan to December 2023. All site teams nominated one or two representatives to attend the meeting and UW coordinated the meeting and together we wrote the EFGH statistical analysis plan which is available on clinicaltrials.gov. The co-development of this plan forced us to think through critical data decisions that would impact data analysis and interpretation ensuring that those decisions are transparent and not made in a silo.

As results were developed the manuscript working groups served as a forum for data interpretation and collaborative decision making for how to best present the data analyses in the context of each manuscript.

d) Manuscript preparation:

To prepare for primary manuscript working groups (described below), the central coordination team, with input from site PIs and other coordinating bodies, developed the EFGH Consortium Authorship & Publication Charter. The purpose of the 18-page Charter was to define Consortium outputs, outline roles and responsibilities, and establish shared principles and procedures surrounding the publication process by which all EFGH Consortium members wishing to participate in Consortium outputs will adhere to, and operate through. This Charter helps to ensure the primary manuscript writing processes remain in alignment with overall Consortium goals and principles related to manuscript writing and results dissemination.

All primary results manuscripts generated by the EFGH Consortium, including this manuscript, were co-written using a working group model grounded in clearly articulated and agreed upon author expectations and roles and responsibilities (as outlined in the Authorship & Publication Charter referenced above). Each primary paper had authorship representation from all sites and coordinating bodies and included 9 co-first, between 16-56 middle authors, and 9 co-last authors. With this inclusive authorship model, authors were randomized within their specific author groups to distribute authorship order and paper leadership opportunities equally across teams and an opt-in model was used to ensure active buy-in

Co-first and co-last authors from the same site were paired together to establish formal writing support and mentorship relationships. Co-first authors received writing assignments for different parts of the manuscript that were then drafted, reviewed by first-first and last-last authors and collated for unified voice. The coordinating PI was responsible for reviewing all Consortium publication drafts, providing written feedback at various stages, supporting first-first and last-last authors in their leadership of the manuscripts, and harmonization across manuscripts.

Each manuscript working group meeting was hosted on Zoom by UW Coordination and required planning, agenda and slide development, and facilitation preparation and coaching for lead authors. To finalize the papers, lead authors with support from UW coordination, led the co-author review process and led responses to peer review. To maintain transparency throughout the process, regular updates were provided to author groups to keep them informed of progress toward publication and revisions as indicated.

2. How was funding used to remunerate and enhance the skills of researchers in the countries of study? And how was funding used to improve research infrastructure at the study sites?

   Potentially effective investments into long-term skills and opportunities within local institutions could include training or mentorship in analytical techniques and manuscript writing, opportunities to lead all or specific aspects of the study, financial remuneration rather than requiring volunteers, and other professional development and educational opportunities.

   Improvements to research infrastructure could include funding extended trial designs (e.g., platform trials), establishment of long-term contracts for research staff, building research facilities, and setting up local control of funding allocation.

We used EFGH funding to build capacity across the consortium through:

<u>eDGH Course Offerings:</u> Site investigators (between 4-6 course participants per site) were provided with multiple opportunities to participate in University of Washington Global Health E-Learning courses (eDGH), including: Introduction to Epidemiology for Global Health, Leadership and

Management in Health, and Fundamental of Global Health Research. These remote learning courses were very well attended with representation from each of the 7 EFGH sites at all 3 courses and covered highly relevant content and provided Consortium Investigators across all levels opportunities to participate in formalized training opportunities to cite in CVs, fulfil institutional continuing education requirements, and build strong content expertise.

Rising Star Seed Award & Early Career Peer Mentorship Cohort Planning: Grant/manuscript writing, data analysis, and independent funding were identified as the highest-ranking goals of Consortium members in the Site Goals surveys. To respond to these partner-specified goals, the UW Coordination team and BMGF collaborated to develop two programs to support junior low- and middle-income country (LMIC)-based investigators in their research career development. The *Rising Star Seed Award* supported the research career pathways of 7 (one from each site) highly talented junior investigators from EFGH partners institutions. These awards were all in the amount of $20,000-$30,000 to support independent research projects that leveraged the EFGH data and infrastructure. *The* Manuscript Writing Cohort aimed to promote the use and first-author publication of existing EFGH data by junior LMIC researchers within the Consortium. This 16-month mentorship and training program provided didactic training, project matching (if desired), and one-on-one mentorship guiding participants through the process of proposing, writing, and publishing on a research question addressable with existing EFGH data. These activities were described in a summary article of EFGH found here: https://pubmed.ncbi.nlm.nih.gov/38532964/ (Vannice et al., Open Forum Infectious Diseases, 2024)

Research infrastructure:
UW Coordination team partnered with the funder to develop a plan for a 'Research Administration Assessment.' This assessment was a component of the collective effort to critically evaluate funding streams and funding mechanisms as part of a commitment to decolonizing global health and to responding to sites' goal of receiving direct funding from funders. This assessment was intended to help us understand the administrative and fiscal systems that support an organization's ability to receive project funding directly from a funding organization, such as the Gates Foundation and the rationale behind why some organizations may prefer to work with an intermediary institution to receive funding.

The UW and KEMRI decided to enter into a direct subcontract agreement to implement Phase B of the study. While this was not direct funding, it eliminated the pass through via UMB, and reduced administrative needs and streamlined communication and coordination. In the initial months of Phase B, the UW and MRC The Gambia also decided to create a direct subcontract agreement starting with year 2 of that grant. This model of "more direct" funding is welcome by the sites and has numerous benefits, including faster timelines for funds to be released, fewer indirect costs, and more streamlined coordination and accountability.

During the coordination visits, in-person partner assessment occurred at the Kenya and the Gambia sites. These activities included one-on-one meetings with budget and grant administrators, as well as meetings with the PI(s) and relevant scientific staff to understand programmatic issues/challenges related to funding streams.

By the third phase (C) of EFGH all sites (including the UW) were awarded funding directly to continue the work of the EFGH study to continue to develop manuscripts, disseminate fundings and further understand policymaker priorities among other objectives set by sites with the Gates Foundation directly. This demonstrates a clear commitment to redirecting the flow of funding while maintaining a strong collaborative relationship with global partners.

3. How did you safeguard the researchers who implemented the study?

Please describe how you guaranteed safe working conditions for study staff, including provision of appropriate personal protective equipment, protection from violence, and prevention of overworking.

Internal and external (by Westat) monitoring occurred twice yearly as well as before study initiation.

These visits included observation of all study activities in accordance with GCP/GCLP. Each EFGH implementing team was led by the site's primary investigator who was responsible for study oversight and all HR-related issues. Contractual agreements between the University of Washington and sub-contracting institutions include language about following local employment laws and regulations.

Benefits to the communities and regions of study

4. How does the study address the research and policy priorities of its location?

How were the local priorities determined and then used to inform the research question? Who decided which priorities to take forward? Which elements of the study address those priorities?

The EFGH study was preceded by large diarrhea etiology studies, the Global Enterics Multicenter Study (GEMS) and the Etiology, Risk Factors, and Interactions of Enteric Infections and Malnutrition and the Consequences for Child Health and Development Study (MAL-ED) and a large clinical trial (Antibiotics for Children with Severe Diarrhea [ABCD]). These studies highlighted *Shigella*'s critical role in diarrhea and linear growth faltering and really emphasized the critical role of antibiotics for averting bad outcomes from *Shigella*. EFGH study sites also participated in these previous studies. Simultaneously, Shigella vaccines have seen renewed development interest which led to important qualitative work understanding national stakeholders and health care providers in high-burden countries evaluating the prioritization of *Shigella* vaccines (Fleming et al., Vaccine X, 2023). This work revealed that the relative importance of Shigella vaccines compared to other interventions increases dramatically when a country considers antimicrobial resistance and Shigella's unique association with linear growth faltering, a precursor of stunting. This motivated EFGH and its aims to establish local data on the burden, consequence, cost, of *Shigella* diarrhea in 7 country sites in Africa, Asia, and Latin America, countries likely to be early adopters of eventual licensed *Shigella* vaccines. EFGH sites will be ready to quickly implement rigorous and efficient vaccine trials and provide critical data to policy makers about the relative importance of this vaccine-preventable disease, accelerating the time to vaccine availability and uptake among children in LMICs.

5. How will research products be shared in the community of study?

For instance, will you be providing written or oral layperson summaries for non-academic information sharing? Will study data be made available to institutions in the region(s) of study?

The Lancet Global Health *encourages authors to translate the summary (abstract) into relevant languages after paper editing; do you intend to translate your summary?*

The active study implementation phase of EFGH concluded in May of 2025 and at the same time we launched the next 2 year phase, which focuses on results dissemination. A dissemination toolkit was developed with input from consortium members and provided to them for adaptation to their local context. This includes the following:

1. Policy brief
2. Plain language summary
3. Technical summary
4. Infographics
5. Slide decks

One globally focused webinar has already been hosted by the UW coordination team which included speaker representation from each site and can be viewed here: https://www.youtube.com/watch?v=ZhrS3oAVOLc

We also intend to translate the summary of this manuscript in to French, Spanish, and Urdu which were identified as the languages that would reach most broadly across the seven EFGH sites.

6. How were individuals, communities, and environments protected from harm?

a) *How did you ensure that sensitive patient data were handled safely and respectfully? Was there any potential for stigma or discrimination against participants arising from any of the procedures or outcomes of the study?*

Protocols, consent forms, and questionnaires underwent ethical review at all EFGH institutions. Consent forms were translated in local languages and identifying information stored in locked cabinets accessible only to designated study staff. All study team members underwent human subjects protection training and training certificates were reviewed for recency during external monitoring visits.

b) *Might any of the tests be experienced as invasive or culturally insensitive?*

Sample collection (rectal swabs and dried blood spots) are invasive and caregivers were counselled on these procedures and only those who consented were enrolled. In some settings, rectal swab collection was not as prominent and teams experienced some concerns from the community stemming from pre-existing notions and stigma. Study teams conducted additional community sensitization activities about rectal swab collection to increase acceptability and thoroughly explain the procedures before and during sample collection to the participant's caregiver.

c) *How did you determine that work was sensitive to traditions, restrictions, and considerations of all cultural and religious groups in the study population?*

Manuscript working groups served as an important time and place to convene on this, and having continuous site representation when designing the procedures, ensured the work was

aligned with setting-specific cultural considerations. Sites were also empowered to adapt central SOPs to better reflect setting/cultural nuances

d) *Were biowaste and radioactive waste disposed of in accordance with local laws?*

Yes, each site was responsible for complying with local laws and policies related to appropriate disposal requirements for any biowaste associated with the study.

e) *Were any structures built that would have impacted members of the community or the environment (such as handwashing facilities in a public space)? If so, how did you ensure that you had appropriate community buy-in?*

NA

f) *How might the study have impacted existing health-care resources (such as staff workloads, use of equipment that is typically employed elsewhere, or reallocation of public funds)?*

Study staff wages and benefits were paid by grant funds, and site PIs were expected to account for and staff the project appropriately. Backup stocks of antibiotics and zinc, which are part of diarrhea management/standard of care, were purchased by the study when indicated to mitigate facility stock-out challenges. Lab supplies and reagents for project-related lab procedures were also provided.

7. Confirm that local ethics review was sought, and please provide the approval number. If not sought, please explain why.

This study was conducted according to Good Clinical Practice (GCP), including Good Clinical Laboratory Practice (GCLP), the Declaration of Helsinki, IRB and local rules and regulations specific to each EFGH country. This protocol was subject to ethical approval from the Institutional Review Boards (IRBs) at each EFGH site.

Bangladesh:
The Institutional Review Board of International Centre for Diarrhoeal Disease Research, Bangladesh gave ethical approval for this work [Approval #: PR-21114]

Kenya:
The KEMRI Scientific and Ethics Review Unit of the Kenya Medical Research Institute gave ethical approval for this work [Approval #: PROTOCOL NO. KEMRI/SERU/CGHR/403/4362]. Kenya Medical Research Institute, has been licensed by The National Commission for Science, Technology and Innovation to conduct research as per the provision of the Science, Technology and Innovation Act, 2013 (Rev.2014) in Siaya on the topic: ENTERICS FOR GLOBAL HEALTH: SHIGELLA SURVEILLANCE STUDY (EFGH) for the period ending : 27/January/2024 [License No: NACOSTI/P/22/15379 & NACOSTI/P/23/23350].

Malawi:
The College of Medicine Research Ethics Committee of Kamuzu University of Health Sciences gave ethical approval for this work [Approval #'s: P.10/21/3437]. The Central University Research Ethics Committee D of the University of Liverpool gave ethical approval for this work [Approval #: 10596].

Mali:
Universite Des Sciences, Des Techniques et des Technologies de Bamako gave ethical approval for this work [Approval #'s: 00000918, 00000964, 0000091, 00000189, 00000440, 0000022, 00000383, 00000279]. The University of Maryland, Baltimore Institutional Review Board gave ethical approval for this work [Approval #'s: HP-00098210, HM-HP-00098210-1, HM-HP-00098210-2, HM-HP-00098210-3].

Pakistan:
The Ethics Review Committee of The Aga Khan University gave ethical approval for this work [Approval #'s: 2021-6932-19680, 2022-6932-21888, 2022-6932-23332, 2022-6932-23399, 2023-6932-24228, 2023-6932-25760]. The Pakistan National Institutes of Health, Health Research Institute, National Bioethics Committee gave ethical approval for this work [Approval #'s: No.4-87/NBC-746/22/1556 , No.4-87/NBC-746/22/160, No.4-87/NBC-746-Exten/23/1577].

Peru:
Comité Institucional de Ética en Investigación Prisma gave ethical approval for this work [Approval #'s: CE0043.22, CE0669.23, CE0674.22, CE0513.23, CE0669.23]

The Gambia:
The Observational / Interventions Research Ethics Committee of the London School of Hygiene & Tropical Medicine gave ethical approval for this work [Approval #: 26515].

U.S. Coordination:
The Human Subjects Divisions of The University of Washington, Seattle, USA, The University of Virginia, Charlottesville, USA, and Emory University, Atlanta, USA, determined that the activities of the coordinating bodies do not constitute human subjects research as defined by federal regulations. Therefore, review and approval by these IRBs was not required.

Secondary analyses

8. Have the data analysed in your study been extracted from another source, such as a national survey, rather than being directly collected by the authors of this paper?

No

If the authors of this paper were not involved in data collection, how were the findings interpreted with sufficient contextual knowledge?
The Lancet Global Health *believe contextual understanding is crucial for informed data analysis and interpretation.*

The authorship model of this paper, by including a large number of junior and non-PI level first and last co-authors, was a way to account for inclusion of perspective across the hierarchy of study teams. Lead authors of this paper, if not directly involved in data collection, were in close

contact with members of their site study teams to provide local contextual expertise to aid in interpretation.

---

9. Finally, please provide the title (e.g., Dr/Prof, Mr/Mrs/Ms/Mx), name, and email address of an author who can be contacted about this statement.

Name: Dr. Patricia Pavlinac, PhD
Email: ppav@uw.edu
